# Dirty engineering data-driven inverse prediction machine learning model

**DOI:** 10.1038/s41598-020-77575-0

**Published:** 2020-11-24

**Authors:** Jin-Woong Lee, Woon Bae Park, Byung Do Lee, Seonghwan Kim, Nam Hoon Goo, Kee-Sun Sohn

**Affiliations:** 1 Faculty of Nanotechnology and Advanced Materials Engineering, Sejong University, Seoul, 143-747 Republic of Korea; 2 Department of Printed Electronics, Sunchon National University, 291-19 Jungang-ro, Sunchon, Chonnam 540-742 South Korea; 3 Advanced Research Team, Hyundai Steel DangJin Works, DangJin, Chungnam 31719 Republic of Korea

**Keywords:** Materials science, Mathematics and computing

## Abstract

Most data-driven machine learning (ML) approaches established in metallurgy research fields are focused on a build-up of reliable quantitative models that predict a material property from a given set of material conditions. In general, the input feature dimension (the number of material condition variables) is much higher than the output feature dimension (the number of material properties of concern). Rather than such a forward-prediction ML model, it is necessary to develop so-called inverse-design modeling, wherein required material conditions could be deduced from a set of desired material properties. Here we report a novel inverse design strategy that employs two independent approaches: a metaheuristics-assisted inverse reading of conventional forward ML models and an atypical inverse ML model based on a modified variational autoencoder. These two unprecedented approaches were successful and led to overlapped results, from which we pinpointed several novel thermo-mechanically controlled processed (TMCP) steel alloy candidates that were validated by a rule-based thermodynamic calculation tool (Thermo-Calc.). We also suggested a practical protocol to elucidate how to treat engineering data collected from industry, which is not prepared as independent and identically distributed (IID) random data.

## Introduction

Recent materials science research has spotlighted machine learning (ML) techniques that involve deep learning (DL)^1–11^. In particular, metal alloy design would be one of the most suitable research areas for DL. A reliable DL model for quantitative material conditions and performance relationships (QCPR) would be the prerequisite for a successful alloy design. To guarantee a successful DL-based QCPR model it is necessary to secure a sufficient amount of training data, but the lack of real-world data in metallurgy research is a serious concern. Therefore, it is customary to use synthetic data to compensate for the dearth of real-world data. Synthetic data are computer-generated using theoretical simulation tools for thermodynamics, molecular dynamics, ab initio quantum mechanics, etc., which have been systematically collected and stored in well-known databases such as NOMAD, AFLOW, OQMD, and Materials Project^12–15^.

Although real-world data-driven ML should work in parallel with simulated data-driven ML, the amount of obtainable real-world data is lacking. A further complication is that the acquired real-world data exhibit many problems having to do with non-identically-distributed, non-curated, and highly-biased status. In addition, although almost all simulated data-driven ML approaches are easily supported by independent identically distributed (IID) random training data, which was the case that we prepared more than 800,000 synthetic XRD patterns in IID random status^16^, it would be nearly impossible to prepare IID random real-world engineering data due to expert intervention while the data are being produced. The present investigation deals with a dataset collected from the industry, consisting of alloy compositions, processing conditions, and tensile properties for 5473 thermo-mechanically controlled processed (TMCP) steel alloys. The data have been collected by the Hyundai steel Co. for the past few years. One of the major aims in the present investigation was to introduce a reasonable protocol to handle this sort of real-world, non-IID dataset collected from an industry.

Several data-driven alloy design strategies are in existence. Metaheuristics along with the experimental evaluation of instantaneous objective function values is also a promising alloy design strategy. Over the past decade we have successfully discovered many inorganic functional materials using metaheuristics strategies^17–19^, but this approach has never been used for metal alloy design. Another promising data-driven approach to metallic alloy design is the reinforcement learning (RL) technique. Since an instantaneous supply of a huge amount of data is necessary for RL, simulator-based data generation would be preferred over an experimental data production platform^20^. The present investigation deals with the most common QCPR models with a fully labeled dataset, such as deep neural network (DNN)^21^, k-nearest neighbors (KNN)^22^, random forest (RF)^23^, support vector machine (SVM)^24^, and Gaussian process regression (GPR)^25^, because these are considered a prerequisite for the major focus of the present investigation—inverse design.

ML-based inverse design has attracted a great deal of attention for use in materials discovery^26–30^. The inverse design we were particularly interested in defines the prediction for an alloy composition, as well as the processing conditions required to accomplish a desired performance for a specific material. For example, if we wanted to produce a steel alloy with a tensile strength and yield strength exceeding a certain level, the inverse ML model would answer the question of which alloy contents and processing conditions should be adopted. DNN never allows their reversible use, because a DNN is not a reversible function. A conventional DNN has a large number of input features that designate the composition and processing conditions of a particular material, but there are much fewer output features that designate material performances. Therefore, an inverse architecture would seem to be nonsensical, although a successful inverse architecture has very recently been reported as a very unusual case^26^. It would never be allowed in a general supervised learning case, although the inverse DNN architecture could be interpreted as a major part of unsupervised learning algorithms such as a variational autoencoder (VAE)^29–33^ and a generative adversarial network (GAN)^34–37^. The inverse prediction is also impractical for other traditional ML approaches such as K-nearest neighbor (KNN)^22^, random forest (RF)^23^, support vector machine (SVM)^24^, Gaussian process regression (GPR)^25^, etc. Consequently, the inverse design is intractable in the current status of ML targeting the deterministic mapping. An enormous time/cost-consuming enumeration strategy based on the conventional forward ML (or DL) model is the only possible way to make an inverse prediction. It should, however, be noted that a so-called combinatorial explosion problem would never allow for the enumeration strategy in most cases.

The aim of the present investigation is to provide a solution for inverse design even when the data quality does not meet a high standard. For this sake, two inverse design approaches were independently implemented. One approach involves a metaheuristics-assisted inverse prediction using a conventional forward DNN model, and the other approach depends on plausible input data generation via a so-called modified variational auto-encoder (MVAE). The metaheuristics is a non-gradient-driven global optimization strategy that includes genetic algorithm^38–42^, particle swarm optimization^43–45^, simulated annealing^46,47^, tabu search algorithm^48–50^, etc. We employed an elitist-improved non-dominated sorting genetic algorithm (NSGA-II)^40^ to inverse-predict desired input solutions using a fully trained forward DNN model. The inverse-prediction in this case represents the optimization of a fully trained ML (or DL) model by taking input data for ML (or DL) model as decision variables and output data as objective functions for NSGA-II. Although there have been several attempts to combine ANN and GA^29–31^, our approach differed from these in that these previous approaches were only concerned with single objective optimization problems based on a shallow ANN architecture with a single output feature along with a limited amount of training data, but our approach is based on multi-objective optimization using a deep ANN architecture (DNN) with multi-output features along with an extensive training dataset.

The second approach includes a modified variational auto-encoder (MVAE)^32, 33^. A similar encoder-decoder approach has been used for a molecular inverse design that uses the combination of a DNN (encoder) and a recurrent neural network (decoder)^28^. The MVAE approach sharply differs from the conventional auto-encoder-based approach in that the MVAE version is not based on deterministic one-to-one mapping. The MVAE focuses more on the generation of plausible data distribution—so-called a probabilistic generative model. The MVAE never aims at a deterministic input–output pair matching but instead plausible input data are generated stochastically. The inverse prediction can be achieved via input data generation on the condition that the generated input data is likely to correspond to desirable output data.

As a result of the NSGA-II and MVAE approach, we obtained 100 alloy candidates, which were concurrently suggested by both the approaches. In other words, the suggested candidates are those that approximately overlap both the NSGA-II and MVAE prediction results. These novel thermo-mechanically controlled processed (TMCP) steel alloy candidates were validated using a rule-based thermodynamic calculation tool (Thermo-Calc.)^51^.

## Results and discussion

### Forward and inverse DNN architectures

The simplest idea for the inverse design model is to set up an inverse DNN architecture by switching input and output layers. However, such a naïve approach would never work, because of the dimension difference between input and output features. When an inverse DNN is thought of as a sort of mapping function that deterministically matches a specific input value with a feature to a corresponding output vector with many features, its inverse architecture would not make sense at all since a variety of output values should be mapped from a certain single input value. One might be misunderstood that Bayesian neural network^52^ would work out as an inverse architecture since it does not give a deterministic output prediction but a distribution of predictions originating from the parameter distribution. However, this sort of Bayesian probabilistic prediction still resides within a narrow range so that it can never be regarded as a desirable inverse prediction method.

**Figure 1:**
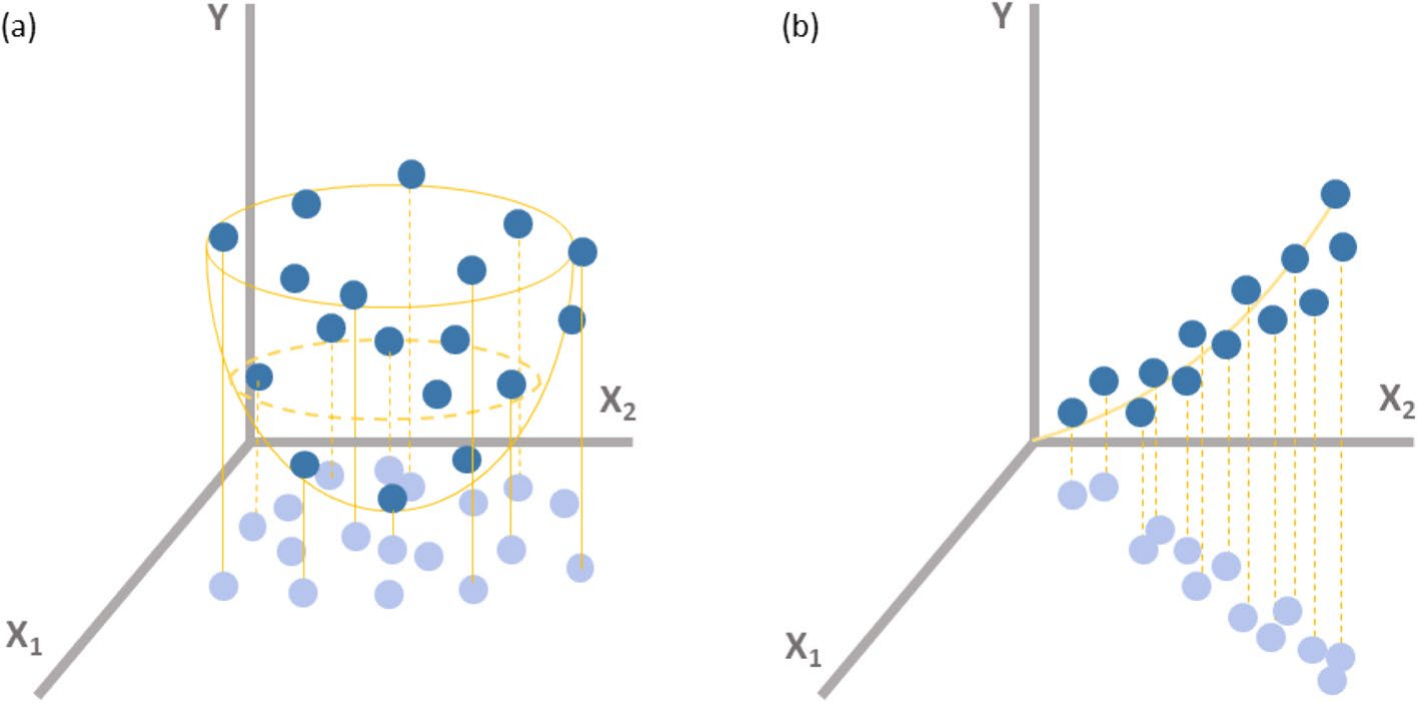
Data distributions along with simple ML models consisting of two input features (x_1_ and x_2_) and an output feature (y). (**a**) A more general data distribution available only for a forward ML prediction model, the parabolic surface represents a forward ML model. No correlation between x_1_ and x_2_. (**b**) A very specific data distribution case, wherein both the forward and inverse ML prediction models are available, the thin solid curve represents both the forward and inverse ML models. Due to a strong correlation between x_1_ and x_2_, either of them could be considered substantially non-existent. The data projection on the x_1_–x_2_ plane, marked in tansparent blue, shows the IID random status in (**a**) and the severe correlation in (**b**).

The intractability of simple (naïve) inverse DNN architecture is also related to the data distribution. Despite a certain dimension difference between the input and output features, the naïve inverse architecture would seem appropriate sometimes. Figure 1a shows a representative example of data distribution for a simple dataset with two input features constituting an IID random distribution and an output feature, and Fig. 1b shows an extremely rare case of data distribution that can allow for a simple inverse-architecture DNN model. It is unfortunate, however, that our dataset seems to resemble that shown in Fig. 1a. The data distribution shown in Fig. 1b indicates that there is a strong correlation between input features (the data projection on the x_1_–x_2_ plane), that is, it is unnecessary to involve both the input features but instead either x_1_ or x_2_ would be sufficient to represent the input data. Accordingly, the inverse DNN architecture-available data distribution shown in Fig. 1b is practically non-existent unless deliberately manipulated. This means that we need another more intricate strategy to make it possible to achieve inverse design modeling for our dataset similar to Fig. 1a. A simple inverse DNN architecture must not be used in this case.

### Dataset preparation

In addition to the above-mentioned intractability of the inverse DNN architecture, another practical problem with the inverse design is an inappropriate data status that is highly biased and correlated. It should be noted that most ML (or DL) regression algorithms work well based on the premise that the output loss (the difference between real and model-predicted outputs) should be IID Gaussian random, although it is not necessarily to secure an IID random input data. However, the IID condition would be required to be met when dealing with the inverse design based on the probabilistic generative models. In fact, however, the data acquired from the industry are not IID random in general. Real-world data are highly biased by human intervention while being produced either in industry or in a laboratory. As a result, some input features exhibit distributions that significantly deviate from the normal distribution. In this regard, we introduced a data reduction process and thereby eventually secured a form of pseudo-IID random input data for use in the inverse design, especially for the probabilistic generative model.

**Figure 2:**
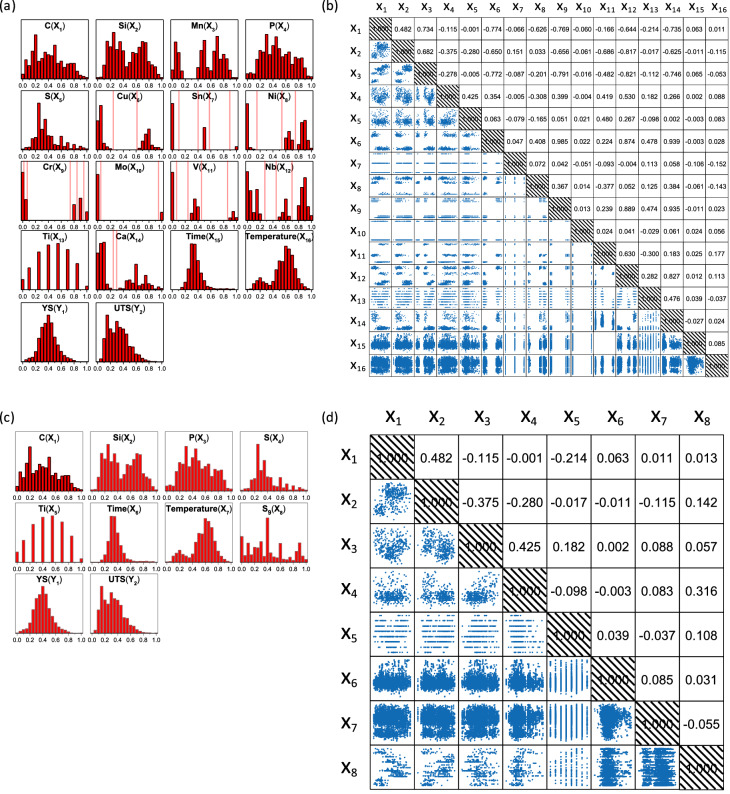
(**a**) 1-D data distribution for each of the 16-input and 2-output features, (**b**) the Pearson correlation coefficient matrix for 16-input features; the upper off-diagonal components are the Pearson correlation coefficients and the lower off-diagonal components are 2-D data distribution plots. (**c**) 1-D data distribution for each of the 8-input and 2-output features, (**d**) the Pearson correlation coefficient matrix for 8-input features.

We collected 5473 alloy entries with 14 alloy components, 2 processing variables, and two materials properties such as yield strength (YS) and ultimate tensile strength (UTS). X_1–14_ are elemental compositions for C, Si, Mn, P, S, Cu, Sn, Ni, Cr, Mo, V, Nb, Ti, and Ca. X_15_ and X_16_ represent heating time and temperature, respectively. This amounted to 16 input features and 2 output features. The input features were min–max normalized such that each feature ranged from 0 to 1, and the output features were standardized such that the mean was 0 and the variance was 1. The distribution of each feature (variable) was plotted as shown in Fig. 2a. The output features (YS and UTS) can be approximated to Gaussian distribution. It is obvious, however, that several of the input features could not be approximated to the continuous random variable. Because the data production process involved a certain degree of biased human intervention, the acquired data were neither continuous nor IID random. Some of the continuous variables were not in a unimodal normal distribution, but were, instead, in the form of multi-modal distributions, and the discrete variables were either in distributions similar to Binomial and Poisson distributions or even in a distribution similar to a Bernoulli distribution. Although there are some systematic ways of mapping data from various distributions to a normal distribution, e.g., Box-Cox^53^, Yeo-Johnson^54^, and Quantile transformations^55^, we did not incorporate them in the present investigation, since the data discretization methodology that we adopted outperformed them.

The inter-correlations between the input features were also examined in terms of the Pearson correlation coefficient. The schematics (so-called Pearson correlation matrix) for 2-D data distribution and Pearson correlation coefficients for every possible input variable pair appear in Fig. 2b. The data distribution was not continuous IID random, and was discrete and highly biased. This sort of ‘dirty data’ could never be avoided when raw data are collected from an industrial setting. We did not employ the principal component analysis (PCA)-based data reduction since the ‘dirty data’ trait survived even in the PCA-reduced lower dimensional space.

The use of an intact raw dataset (16 input and 2 output features) appears to be inefficient for the inverse design based on the probabilistic generative model, since it is not IID-random. However, we employed 16 input and 2 output features for the conventional forward-prediction DNN model by maintaining the intact, raw state of the industrial data. Since one of the most prominent merits of deep learning is ‘the use of raw data with no handcrafted feature engineering’, it is unnecessary to employ the preliminary data-dimension-reduction process. In fact, some of many hidden layers in the DNN architecture could be responsible for a possible data dimension reduction, as was the case that single hidden-layer auto-encoders have proven equivalent to a typical PCA^56, 57^. Accordingly, there should be no need for feature engineering such as PCA prior to the DNN training, as far as a conventional forward prediction model is used. Other forward prediction models such as KNN, RF SVM, and GPR also worked for the raw industrial dataset. However, MVAE never worked for the raw industrial dataset. Therefore, a special measure should be taken to constitute a dataset exhibiting IID-random normal distribution, which could be used for the MVAE-based inverse prediction model. More details about the data reduction process are discussed in the Method section.

### Conventional forward DNN models for 16- and 8-input features

**Figure 3:**
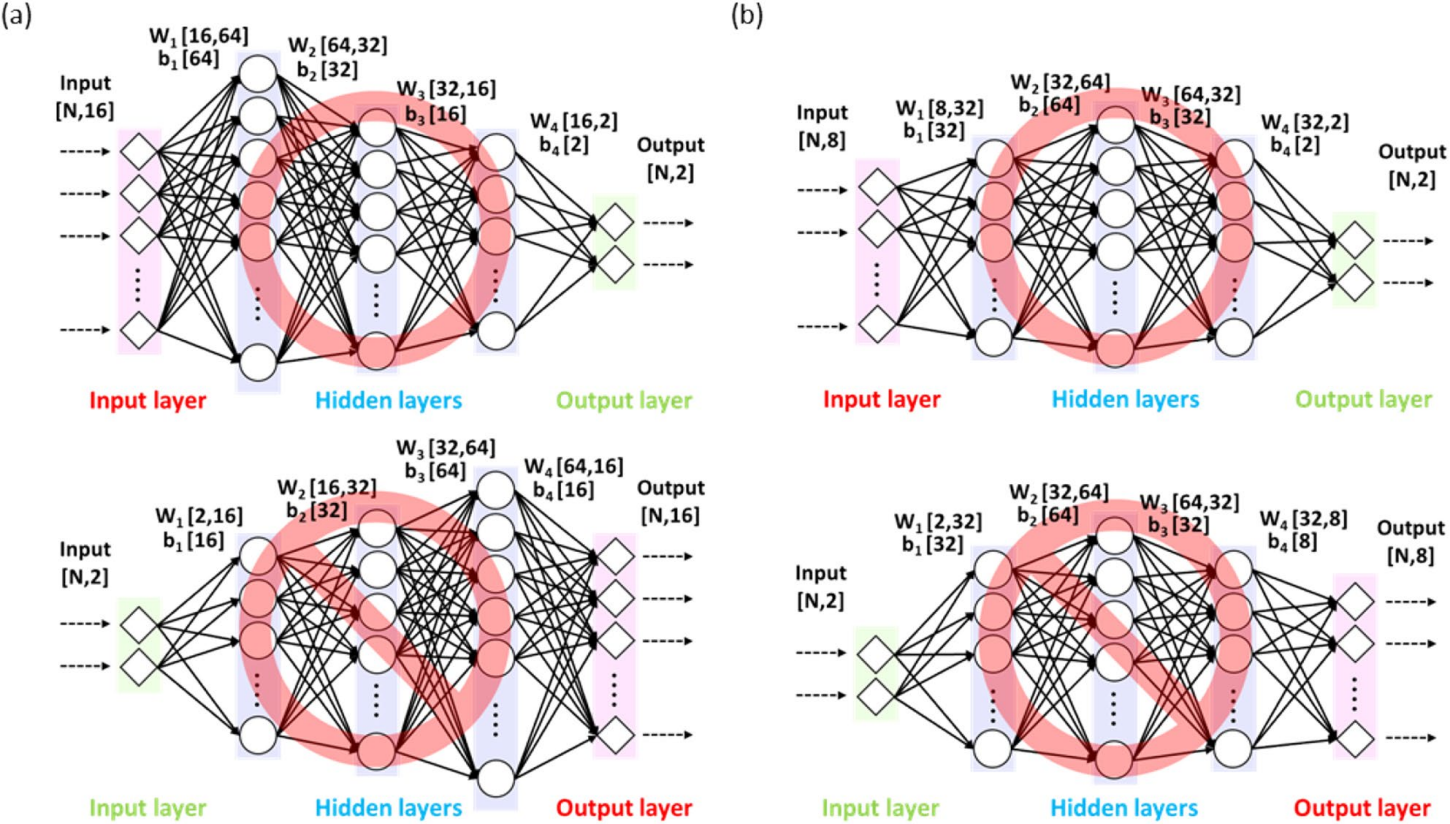
(**a**) The DNN architecture selected for 16 input features, and (**b**) for 8 input features, (top) along with inverse architectures that could never succeed (bottom).

**Figure 4:**
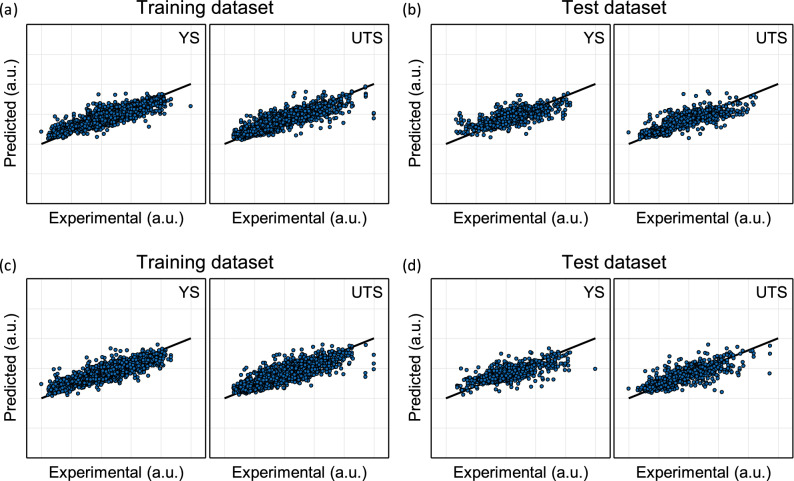
The predicted output versus the real output for (**a**) the training dataset, (**b**) the hold-out test dataset for a 16-input-feature DNN, (**c**) the training dataset, and (**d**) the hold-out test dataset for an 8-input-feature DNN.

We set up 60 different DNN architectures for the 16-input-feature DNN and tested them individually. All the architectures are thoroughly described in Supplementary Table S1a. The architecture selection process did not seem to be as promising as the recently spotlighted Bayesian optimization^58^ that has been widely utilized in the hyper-parameter tuning process. Since the test MSE was not highly dependent on the DNN architecture among the 60 candidates as evidenced in Supplementary Table S1, however, the test of this limited number of architectures was sufficient to tentatively pinpoint the best one. From the architecture selection procedures, we finally adopted a three-hidden-layer-architecture with (16):64:32:16:(2) nodes per each layer, as shown in Fig. 3a. The dropout and/or batch normalization did not take effect for the best architecture. The training was executed by employing a fivefold-cross-validation strategy and thereafter a test was implemented using a holdout dataset that was not used for the training process. The MSE and R^2^ values for the hold-out test dataset are listed in Supplementary Table S2. The predicted YS and UTS were plotted versus the real values for both the training dataset and the holdout test dataset, as shown in Fig. 4a,b.

The reduced 8-input-variable dataset, which can be roughly approximated to an IID-random version (so called pseudo IID-random), was used for alternative DNN training. We also tried 60 different architectures for the 8-input-feature DNN, as shown in Supplementary Table S1b and thereby a three-hidden-layer architecture with (8):32:64:32:(2) nodes per each layer was finally selected, as shown in Fig. 3b. The test MSE for the 8-input-feature dataset was slightly higher than that of the 16-input-feature DNN. The simplified reduction of the original input dataset was the reason for the slight improvement. The predicted YS and UTS was plotted versus the real values for both the training dataset and the holdout test dataset, as shown in Fig. 4c,d. Since the creation of the 8-input-feature dataset originated from the intention to use it for a more advanced inverse design model such as MVAE, the results of this conventional DNN training for the forward prediction should be considered a trial run prior to development of the ultimate inverse design model.

Figure 3 also show failed naïve inverse architectures (two architectures on the bottom). The difference in the number of features between the input and output layers matters in judging whether or not an inverse architecture would work. Inverse architectures with significantly fewer input features than output features could never constitute a promising inverse predictor. In the present study, unfortunately, the difference in the number of features between the input and output layers was significant (2:16 and 2:8) for an inverse architecture. It should be noted that a simple architecture inversion is not a solution for an inverse prediction. We also implemented another forward DNN approach by transforming regression to classification, and the full details are provided in the Supplementary information.

### Other forward ML models for 16- and 8-input features

**Figure 5:**
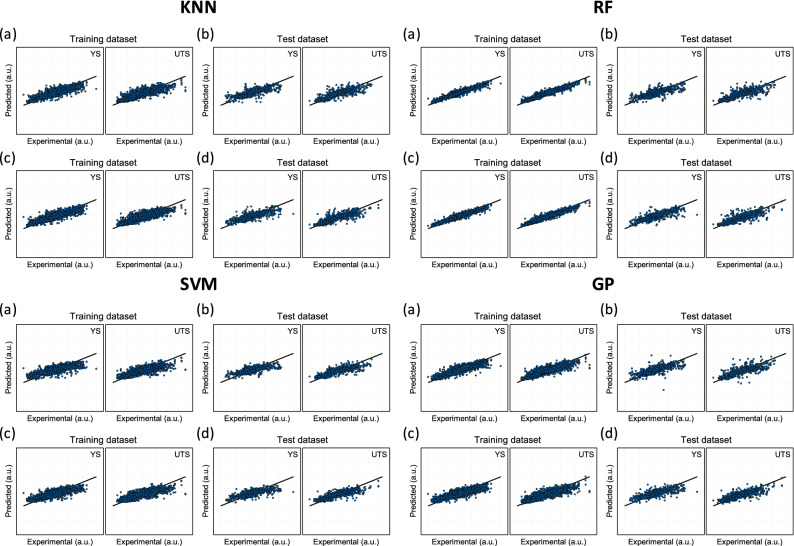
The ML results for KNN, RF, SVM, and GPR. The predicted output versus the real output for (**a**) the training dataset, (**b**) the holdout test dataset for a 16-input-feature ML, (**c**) the training dataset, and (**d**) the holdout test dataset for an 8-input-feature ML.

We employed K-nearest neighbors (KNN)^22^, Random forest (RF)^23^, support vector machine (SVM)^24^, Gaussian process regression (GPR)^25^ along with the DNN approach. Similar to DNN training, both regression and classification were also implemented for the 16- and 8-input feature datasets when the KNN, SVM, RF, and GPR algorithms were employed. The predicted YS and UTS was plotted versus the real values for both the training dataset and the holdout test dataset, as shown in Fig. 5. Supplementary Table S2 shows the results in terms of the test MSE and R^2^ for regression and the test accuracy for classification. The same hold-out test dataset was used for all the algorithms. All the algorithms yielded similar performances. However, a DNN is more favorable from the viewpoint of inverse predictability. Once a DNN is fully trained, the training data are no longer needed to produce new predictions. The training dataset could be replaced by a smaller-sized parameter dataset (weights and biases). This is obviously not the case with some others, and, therefore, basing the traditional ML algorithms on brute force would make the inverse prediction more expensive. This concept is concerned with an issue of whether the ML algorithm of interest is parametric or non-parametric. DNN belongs to the parametric ML algorithm that plays a role in data reduction. However, KNN is a typical nonparametric ML algorithm, in which the entire data set is required every time a new prediction is to be made.

### Inverse prediction based on a non-dominated sorting genetic algorithm (NSGA-II)

**Figure 6:**
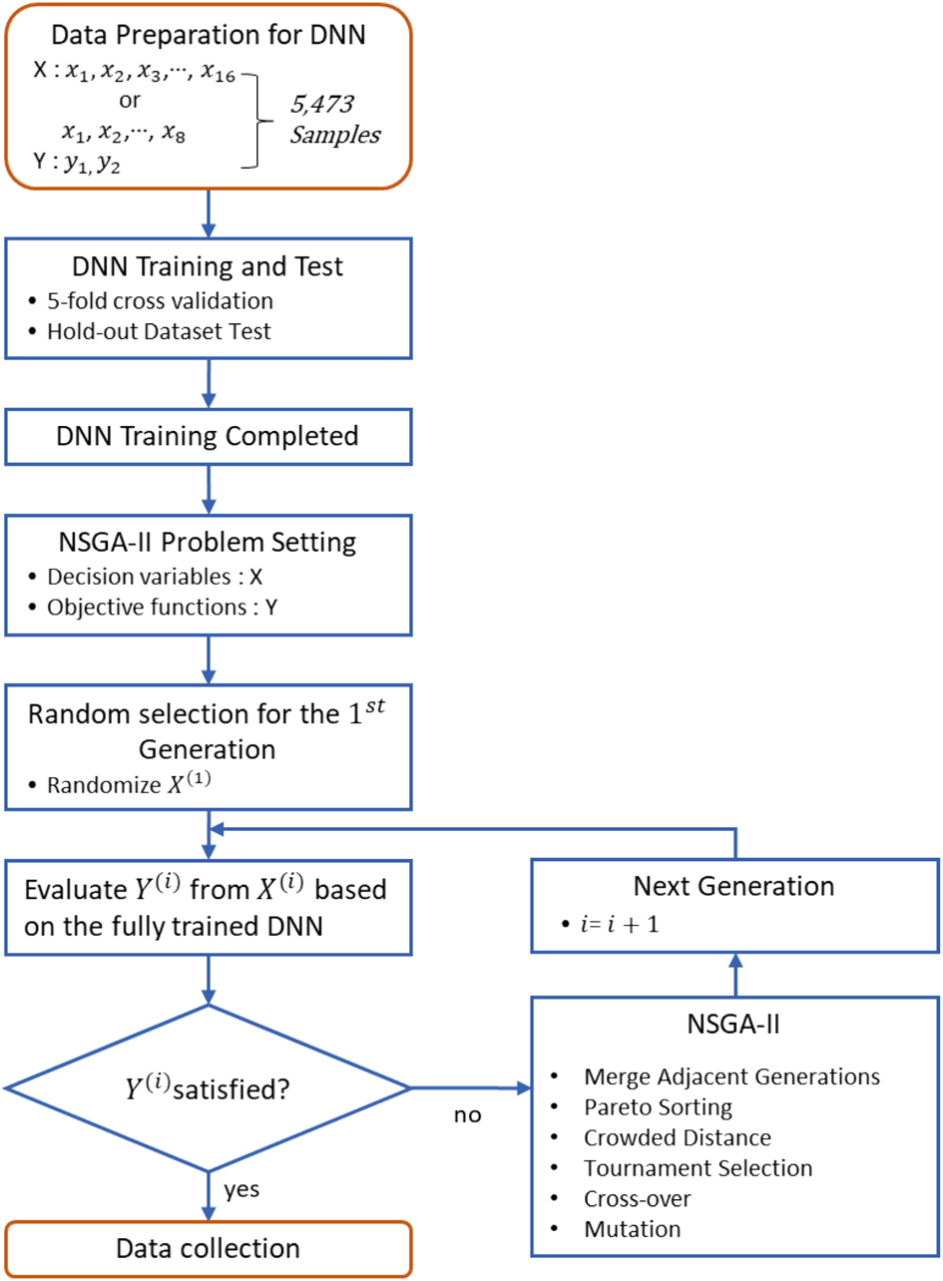
The flow chart briefly describing the NSGA-II algorithm execution.

The NSGA-II was employed for an inverse prediction using fully trained 16- and 8-input feature DNNs. The genetic algorithm (GA) is the most popular form of metaheuristics^38^. The objective function (or fitness function) is either minimized or maximized by adjusting the decision variable based on the principle of natural selection. This sort of artificial evolution was affected by several hyper-parameters controlling selection, mutation, cross-over, and elitism. A more advanced GA would enable multi-objective optimization. The NSGA was developed by employing a Pareto optimality theory, and was improved when a new elitism strategy was introduced^39^. The elitist-improved NSGA, the so-called NSGA-II, was a very versatile multi-objective optimization algorithm for materials discovery^40^, but its performance is restricted to double-objective problems^41, 42^. The NSGA-III was recently introduced to tackle actual multi-objective optimization problems, but we adopted the NSGA-II in the present investigation since only two objective functions existed. A brief schematic of the NSGA-II algorithm-assisted inverse prediction process is given as a flow chart in Fig. 6. A brief introduction is described in Supplementary information, and a more detailed introduction is aptly described in our previous reports^59–61^.

GAs have previously been used in association with ANNs^29–31^. However, single-objective problems were only concerned with single-feature-output ANNs. As mentioned above, neither the NSGA-II nor the NSGA-III has ever been used for an inverse prediction using ANN optimization. The objective functions of our optimization problem were YS and UTS, which are two output features in our DNN, and the decision variables were the 16- or 8-input features. Both of these objective functions should be simultaneously maximized by the NSGA-II iteration. Each NSGA-II execution produced 200 generations with a population size of 50 per each generation. Every NSGA-II execution provided a very narrow Pareto frontier in the last (200th) generation, so that we randomly collected one representative solution from the Pareto frontier in the last generation. We had 100 NSGA-II executions for 16- and 8-input-feature DNNs (50 per each). The solution that we picked up in every single NSGA-II execution differed from one another but occasionally converged on a similar solution. This means that the terrain of the output feature in such a high dimensional (16- or 8-)input-feature space for a fully trained DNN (although it is impossible to visualize) does not provide so many local optima. Accordingly, various NSGA-II implementations could by chance reach the same optimal point.

**Figure 7:**
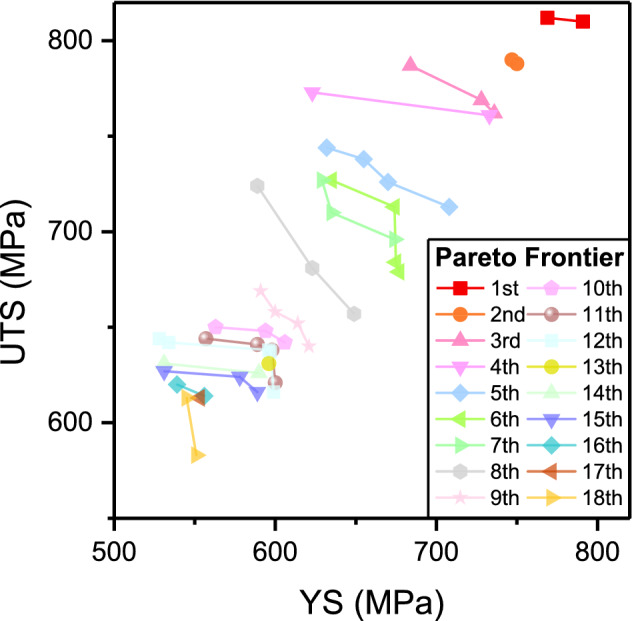
The Pareto-sorted YS and UTS data predicted by the NSGA-II execution. Each Pareto rank is represented in different colors; the first Pareto frontier is in black.

In total, 100 plausible solutions (50 per each of the 16- and 8-input-feature DNNs) were finally obtained from the NSGA-II-assisted inverse prediction process. It has also been noted that some of the solutions from both the 16- and 8-input-feature DNNs are similar, and those from the 8-input-feature DNN were a bit crude since the nine features chosen for the merge were not precisely pinpointed, and only a rough range was actually available. The solutions from the 8-input-feature DNN were decoded into the original 16-input features by adopting the center values for every range, so that a one-to-one transformation could be available between the 16- and 8-input-features. To examine the similarity between the 16- or 8-input-feature DNN solutions, the 2500 norms (50 × 50 distances) between the solutions (input feature vectors) from the 16- and 8-input-feature DNNs were computed and ranked. It should be noted that the rank is based on the solution norm, not on the YS and UTS value. The higher the rank, the smaller the norm. As a result, the top 50 solutions were selected from either the 16- or 8-input-feature DNN solutions in terms of the norm values. This means that the top 25 norms were selected out of 2500, and these pinpointed both the solutions constituting the norm. These solutions could be approximately regarded as an overlap (a.k.a. pseudo-overlap) between the 16- and 8-input-feature DNN solutions and thereafter appointed as the final predictions corresponding to desirable (maximized) YS and UTS. These solutions obtained from the NSGA-II execution will also be compared with those from the other inverse design method, so-called MVAE. Supplementary Table S3 shows the solutions obtained from the NSGA-II executions for both the 16- and 8-input-feature DNNs, which denote the input features (= decision variables), i.e., the alloy compositions and processing conditions. Figure 7 shows the Pareto-sorted YS and UTS data corresponding to the 50 pseudo-overlapped solutions selected from the NSGA-II executions. Unfortunately, however, it was impossible to graphically visualize the solutions (input feature vectors) that were located in the 16-dimensional hyper-space.

### Modified vibrational autoencoder (MVAE)

An alternative inverse design was also attempted by employing a modified variational autoencoder (MVAE). The main function of an autoencoder (AE) is to find hidden features (a latent vector) from which the input data can be reproduced^31^. AE consists of an encoder that reduces the input data dimension to a latent vector with much lower feature dimensions and a decoder that reproduces the input data from the latent vector. The variation autoencoder (VAE)^32, 33^ differs from an AE in that the purpose of AE is to find hidden features to be used for the reproduction of input features, but the VAE aims to generate plausible feature vectors that are not in complete agreement with the input feature vectors but in the same style. For VAE, the hidden feature distribution is approximated to a diagonal normal distribution, N(0, σ^2^·I), or to a standard normal distribution, N(0, I), and the data (Z) sampled from this hidden feature distribution will be used as input for a decoder to achieve plausible data generation. In this regard, the VAE is a probabilistic generative model for plausible data generation based on the unsupervised learning algorithm, whereas a conventional AE is akin to a deterministic one-to-one data mapping that is based on the least square regression process (maximum likelihood estimation) with the same data used for both input and output. In fact, the conventional AE never worked for our datasets (both the 16- and 8-input-feature). We employed the VAE as an alternative to the NSGA-II-assisted inverse prediction with the expectation that it could generate plausible input data that could give rise to a desired set of YS and UTS values. This implies that the inverse prediction could be accomplished using the VAE algorithm.

Instead of the conventional VAE, wherein a hidden feature (Z) distribution is approximated to be either N(0, σ^2^·I) or N(0, I), we modified the conventional VAE in a way that the latent feature distribution can be equated to the real YS and UTS data distribution. The mean and covariance of real YS and UTS distribution are located in the hidden layer in the center of VAE. This indicates that the Z distribution was approximated to the real YS and UTS data distribution that is certainly a non-diagonal Gaussian. We adopted the assumption that the hidden features could be anything as far as the input features could be inferred from them. In a contradiction to the conventional idea that the Z data should be in an arbitrary Gaussian distribution with a diagonal covariance matrix, we equated the Z distribution to the real YS-UTS distribution, which is a Gaussian distribution with a non-diagonal covariance matrix.

**Figure 8:**
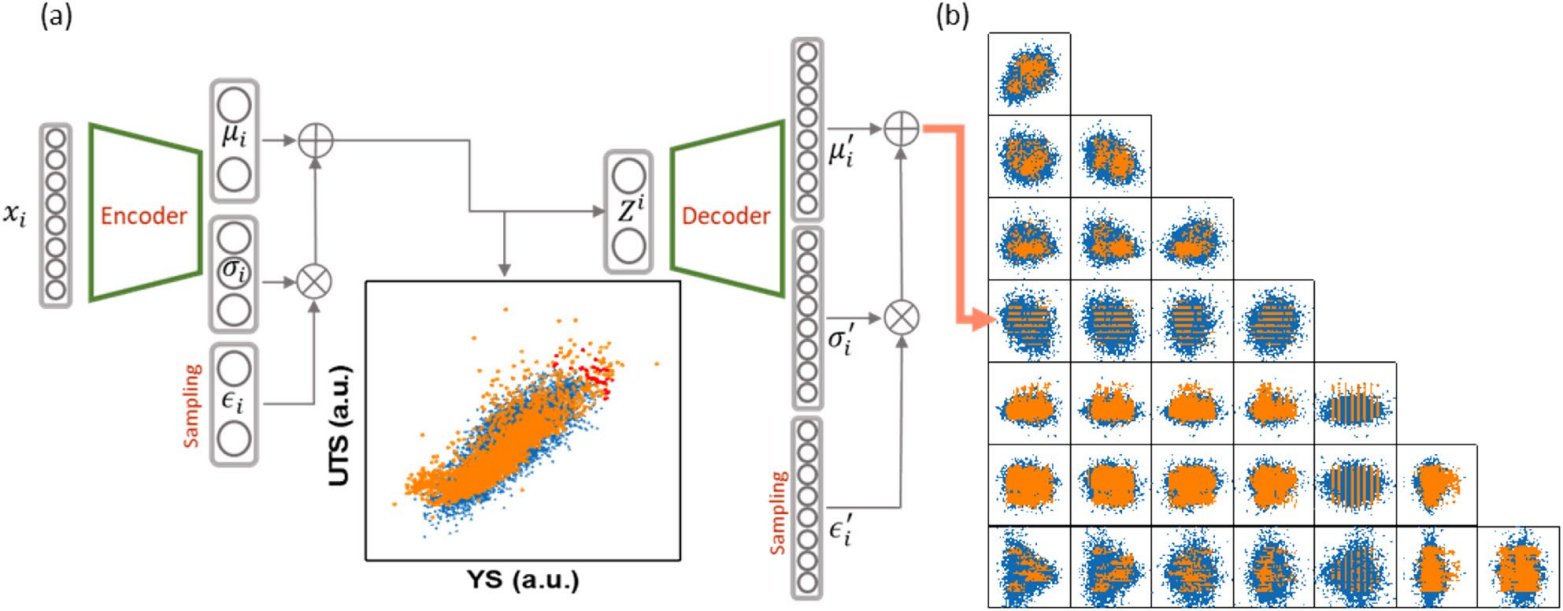
(**a**) The MVAE architecture exhibiting both the encoder and decoder and the distribution of Z data (blue crosses) sampled from the latent vector, along with the real YS-UTS data plot (amber dots), and the red dots in the Z distribution that designate the top four Pareto frontiers to be selected as a decoder input batch for an inverse prediction; (**b**) the MVAE-generated $\widehat{\mathrm{X}}$ data distribution sampled from the decoder output layer (blue crosses) and the input X data (amber dots); the multivariate $\widehat{\mathrm{X}}$ and X data are schematically represented as 2-D data distributions for all possible binary feature e pairs.

The raw data collected from the industry were not suitable for most probabilistic generation algorithms that were designed on the basis of assumptions that the training data would be well-curated IID random data. It is relatively easy to prepare such data when the virtual data were produced using theory-based simulation programs. To the contrary, industry data generally are extremely biased, anomalous, and discontinuous. For this reason, only the 8-input-feature dataset approximately presumed to be a pseudo-IID Gaussian was adopted for MVAE training, and the non-IID 16-input-feature dataset was precluded from the MVAE approach. In fact, when the 16-input-feature dataset was applied to the MVAE, it was never successful. As shown in Eq. (1), the VAE algorithm was developed on the basis of a prerequisite that both the input and Z data constitute multivariate Gaussian distributions. Therefore, only the 8-input-feature dataset is eligible for the MVAE. Figure 8a shows the MVAE architecture exhibiting both the encoder and decoder and the distribution of Z data sampled from the hidden features (= the mean and covariance for Z) along with the real YS and UTS distribution. The loss function responsible for the encoder training is given in Eq. (1).

1 $${\mathrm{D}}_{KL}(\mathrm{N}0\parallel \mathrm{N}1) = \frac{1}{2} \sum_{1}^{N}\left(tr\left({\Sigma }_{1}^{-1}{\Sigma }_{0}\right)+ {\left({\upmu }_{1}-{\upmu }_{0}\right)}^{T}{\Sigma }_{1}^{-1}\left({\upmu }_{1}-{\upmu }_{0}\right)-k+ln\left(\frac{det{\Sigma }_{1}}{det{\Sigma }_{0}}\right)\right)$$

In Eq. (1), D_KL_ stands for the Kullback–Leibler (KL) divergence based on the assumption that both distributions are Gaussian. The KL divergence is a measure of how one probability distribution differs from another^62^. Both the normal distributions, N_0_(μ_0_,**Σ**_**0**_) and N_1_(μ_1_,**Σ**_**1**_), stand for the hidden feature distributions at the encoder output layer (subscript 0) and the real YS vs. UTS distribution (subscript 1), respectively, μ and **Σ** denote the mean vector and covariance matrix, respectively, and k is the dimension of Z (two in our case). The first ${{\varvec{\Sigma}}}_{1}^{{\varvec{N}}}$ on the right side of Eq. (1) does not designate the covariance matrix but stands for a summation symbol with the sample batch size (N). The VAE training led to a minimization of the KL divergence, which means that two normal distributions, N_0_(μ_0_,**Σ**_**0**_) and N_1_(μ_1_,**Σ**_**1**_), should be equated by adjusting the weight and bias of the encoder. The non-diagonal distributions of the real YS and UTS data were represented as a mean vector and a covariance matrix, as shown in Eq. (2).

2 $$\upmu_1 =\left(\begin{array}{c}0\\ 0\end{array}\right),{\varvec{\Sigma}}_1=\left(\begin{array}{cc}1& 0.819\\ 0.819& 1\end{array}\right)$$

Since we adopted standardized YS and UTS data, the mean was a zero vector. The covariance is not a diagonal matrix in sharp contrast to the conventional VAE wherein the covariance matrix is in the form of either a diagonal or an identity matrix. The inset of Fig. 8a also shows the non-diagonal shape of the Z distribution (blue crosses), which is almost coincident with the real YS vs. UTS distribution (amber dots). A sampling method follows the conventional re-parameterization trick that allows for the back-propagation algorithm^32, 33^. Such a good agreement between the Z distribution obtained from the fully trained encoder and the real YS vs. UTS distribution indicates that the training was relatively well done.

The reconstruction loss for decoder was defined as a negative log likelihood, as shown in Eq. (3). The 8-D input features were approximated as a multivariate Pseudo-Gaussian with a diagonal covariance. The decoder output features ${\sigma }_{i,j}$ and ${\upmu }_{i,j}$ constitute an output vector, the dimension of which is twice the input dimension (2J). The reconstruction loss was given as double summations. The first summation for *j* should be done from 1 to the input feature dimension (J), and the second for *i* from 1 to the sample batch size (N). It should be noted that ${\upmu }_{i,j}$ $\mathrm{and }{\sigma }_{i,j}^{2}$ designate the decoder output features for a particular sample *i*, indicating the individual components of mean vectors and covariance matrixes. These differ from those with the same symbols in Eq. (1), which are referred to as the mean vector and the covariance matrix. ${x}_{i,j}$ are also components of the input feature vector.

3 $$\sum_{i}^{N}\sum_{j}^{J}\left(\frac{1}{2} log\left({\sigma }_{i,j}^{2}\right)+\frac{\left({x}_{i,j}^{2}-{\upmu }_{i,j}^{2}\right)}{{\sigma }_{i,j}^{2}}\right)$$

Both the KL divergence loss for regularization and the negative log likelihood loss for reconstruction were minimized simultaneously for training of the entire MVAE. The sum of these terms is referred to as negative evidence lower bound (ELBO). The MVAE produces an 8-D multivariate Gaussian data distribution as an output, such that individual output vectors can be sampled from the mean and covariance nodes that are located on the output layer of the decoder. The MVAE training result in the decoder part was visualized by the 2-D binary distribution for every possible output feature pair, as shown in Fig. 8b wherein the MVAE-generated data ($\widehat{\mathrm{X}}$) distribution sampled from the decoder output layer is marked by blue crosses and the input data (X) is marked by amber dots. The MVAE-generated data distribution and the real input data distribution exhibits an acceptable coincidence as a result of the MVAE training.

The next step is to clarify how to achieve an inverse prediction using the fully trained MVAE. The ultimate goal of the present investigation was to extract alloy compositions and processing conditions that maximize YS and UTS but do not belong to the training dataset. We Pareto-sorted the Z data sampled from the mean and covariance at the center hidden layer (the latent vector) in the fully trained MVAE and pinpointed a small number of higher ranks, which are marked as red dots in Fig. 8a. These selected Z data were inputted to the fully trained decoder, and the $\widehat{\mathrm{X}}$ data sampled from the resultant decoder output could be regarded as a prediction. Because the Z distribution was forcibly equated to the actual YS and UTS distribution during the MVAE training, we appointed only about ten samples belonging to the higher Pareto ranks of the Z data, which suggested higher values for YS and UTS, as input for the fully trained decoder in order to obtain predictions that showed greater promise. Accordingly, the $\widehat{\mathrm{X}}$ data that corresponded to the selected Z data could be regarded as a promising prediction. The selected Z data in every MVAE execution slightly differed from one another due to the sampling process that involved a stochastic choice. In this regard, we repeated a number of MVAE executions and collected all the selected Z and $\widehat{\mathrm{X}}$ data. It should be noted that the MVAE is not a deterministic prediction model but a data generation model in a real sense. However, we demonstrated that the MVAE approach could be used appropriately as a promising predictor in the present study. In practice, the MVAE training was reiterated a huge number of times, and the resultant $\widehat{\mathrm{X}}$ data corresponding to the higher Pareto-ranked Z data were instantaneously collected. As a consequence, we ultimately gathered 1,000 solutions. Most of the inverse-predicted solutions by MVAE executions converged on a relatively narrow range of the solution space, although highly scattered outliers sometimes appeared.

We also suggested an alternative VAE-based inverse design approach using a well-known conditional variational autoencoder (CVAE)^63^. This CVAE approach would seem to be similar to our MVAE approach, but CVAE was not successful as our engineering dataset due to the unclear labeling. In fact, we appointed a Pareto rank as a label by Pareto-sorting the real YS and UTS data. The most simplified labeling scheme was a three-label system that grouped the Pareto ranks into only three classes. Since the inverse-predictability of the CVAE was not as good as the other approaches due to the labeling complication, we skipped the CVAE in this paper, and instead described the CVAE process briefly in the Supplementary information. However, CVAE would work properly if more data were available along with reasonable labeling.

### The final alloy candidates predicted by NSGA-II and MVAE

All the candidates recommended by NSGA-II were compared with those from the MVAE. The NSGA-II produced at best 100 solutions in total because the computational time would have been too long to generate as many solutions as the MVAE produced. We finally pinpointed 50 pseudo-overlapped solutions from the 16- and 8-input-feature DNNs in case of the NSGA-II-based inverse design. By comparison, the MVAE was able to generate an even greater number of solutions in a relatively shorter time frame. Accordingly, we produced 1000 candidates by implementing the MVAE training independently 100 times.

Norms were computed between the NSGA-II and MVAE solutions and we pinpointed 50 MVAE solutions that were closer to the NSGA-II solutions using the same method that was used for the selection of the final 50 candidates from both the NSGA-II-solutions for the 16- and 8-input-feature DNNs. This method is referred to as a so-called ‘pseudo-overlapped-data extraction method’. Supplementary Table S3 shows the final entries that were selected from both the NSGA-II and the MVAE. We did not aim to reckon the relative performance superiority between the MVAE and NSGA-II approaches to the inverse design, but instead, we focused on the coincident result both from the MVAE and NSGA-II. Although these two inverse design approaches come from completely different origins, they ended up with overlapped results. As a result, several plausible solutions were selected from the pseudo-overlapped data by taking into account metallurgy common sense and applying it to theoretical thermodynamic calculations to confirm acceptability.

Instead of the experimental validation that we expect some others who are engaged in the experimental metallurgy to realize, a widely-accepted theoretical computational tool was utilized for the validation of the result. The thermodynamic calculation was performed using the Thermo-Calc. TM TCFE-9 database^51^. The Ae1 and Ae3 temperatures were calculated, and the precipitation reactions of (Ti,Nb)C and VC were evaluated. Such a calculation allowed us to determine whether the alloying compositions could be applied to a typical TMCP process. Prior to thermodynamic calculations, we selected five superior alloys from the final entries obtained through both the NSGA-II and MVAE. The selection criteria were as follows: (1) sufficient alloying elements necessary for strength increase, (2) phase change and precipitate formation in a general hot rolling or heat-treatment process, and (3) control of impurities such as P and S. Supplementary Table S4 shows a group of the best candidate alloys obtained through the above-described selection criteria. The austenite-to-ferrite reaction occurred between 670 and 860 °C, and the precipitation reactions of (Ti,Nb)C and VC were completed at more than 350 °C, which indicates the maximum precipitation. These thermodynamic conditions are quite reasonable in terms of conventional hot rolling and subsequent heat-treatment processes. The increased amount of C, Mn and Nb in the group (in Supplementary Table S4) contributed to improving the strength such that a targeted strength of 800 MPa could be achieved. The impurity levels of P and S were well controlled to match the severe impurity restrictions of API grades.

Besides strengthening by thermodynamic stability of the precipitation, C, Si, and Mn contents of the alloys play a positive role for the TMCP application. Through an appropriate TMCP condition, the alloy would have a typical microstructure, which leads to improved low-temperature toughness. The alloys contain enough Si to inhibit carbides' formation and promote a robust bainitic structure^64^. The C and Mn contents are set lower than in the conventional HSLA alloys, which have 0.10 wt% C and 1.5 wt% Mn. C and Mn are alloying elements essential for securing strength and toughness, but give rise to a severe side effect increasing brittleness when excessively added. It is particularly sensitive to hydrogen embrittlement or stress corrosion cracking due to the segregation band of C and Mn produced through the continuous casting and hot rolling processes. In particular, the band structure containing a large amount of Mn becomes a preferred site for hydrogen. When the hydrogen level increases above a critical point, crack propagation proceeds very rapidly to induce catastrophic failure. Likewise, the segregation zones of C and Mn exhibit very brittle properties against stress corrosion. Usually, the hot rolled steel plate has the segregation in the center region. This region is susceptible to hardening by applying accelerated cooling of TMCP. In a line pipe steel for sour gas containing H_2_S, sulfide stress cracking takes place in the center segregation region. The reduced alloying content of C and Mn is also crucial for preventing the center segregation and subsequent crack and fracture problems^65^. In general, it is hard to find out the alloy composition of 800 MPa strength without significantly increasing the amount of C and Mn. Through the present data-driven searching algorithm, we obtained the outstanding optimum alloy combinations of sufficient strengthening with keeping the C and Mn content below 0.1 wt% and 1.5 wt%, respectively. This implicates a novelty of the suggested alloys.

The improved weldability is another critical factor for alloy design of either HSLA or TMCP steel. The field weldability could be improved by decreasing the value of C_eq. The C_eq value is nominally defined as the following formula.

$${\text{C}}_{{{\text{eq}}}} = {\text{ C }} + {\text{ Mn}}/{6 } + \, \left( {{\text{Cr }} + {\text{ Mo }} + {\text{ V}}} \right)/{5 } + \, \left( {{\text{Ni }} + {\text{ Cu}}} \right)/{15 }\left( {{\text{wt}}\% } \right)$$

The C and Mn contents of our suggested alloys are relatively low compared to the conventional HSLA alloys for TMCP; the C_eq_ values are in the range of 0.3–0.35. The novelty of the suggested compositions is also backed up by the fact that this value is just half of the costs in the traditional TMCP steel products of 800 MPa grade^66^.

## Method

### Dataset preparation details

Rather than typical PCA-based data reduction strategies, we employed an alternative feature engineering technique (data merge or discretization technique), which is heuristic but reasonable. The input features (variables) exhibiting a non-Gaussian distribution (either multi-modal or discrete) were merged such that a resultant, single merged feature could be approximately regarded as a continuous random Gaussian. The non-Gaussian input features (alloy components) such as Mn, Cu, Sn, Ni, Cr, Mo, V, Nb, and Ca were merged. The first step in the merge process was to re-discretize the variables of concern into 2–5 levels. The discretization boundaries are marked as thin vertical lines in Fig. 2a. For instance, the Mo content was simplified as either ‘low’ or ‘high’, and the Cr content was categorized from level_1 to level_5. Consequently, a new merged variable representing the above-mentioned nine variables will be referred to as variable ‘S_9_’. Variable S_9_ was discretized into 3240 (2^3^ × 3^4^ × 5^1^) different levels. However, the total number of available levels for S_9_ was reduced to just 51, since most of the nine constituents were extremely biased, as discussed earlier. This implies that although S_9_ provides 3240 available slots, 5473 data points reside only in 51 slots, indicating that the selected nine original variables were highly concentrated and biased. The creation of the S_9_ variable reasonably distributed over 51-discretezied levels sorted out the non-IID input data problem to a certain extent. Since the remaining seven variables along with S_9_ can constitute a new pseudo-continuous input data space, which can be approximated to a multivariate Gaussian distribution, it could be eventually applied to MVAE-based inverse design. Figure 2c shows the distribution for each of the eight variables including S_9_. Since either discrete distributions or non-unimodal Gaussian distributions remained, this 8-input-feature system can be defined as ‘pseudo-IID’.

Although S_9_ is a discrete variable consisting of only 51 classes, S_9_ could be approximately regarded as a continuous random variable, as with Ti. Moreover, S_9_ could also be roughly approximated to even a Gaussian distribution since this rough approximation would be much more pragmatic than using the severely anomalous distributions of all the previous nine variables. The Pearson correlation coefficient matrix for every possible input feature pair is schematically represented in Fig. 2d. In contrast to the prior case of 16-input features, the correlation between input variables was alleviated and thereby the 8-input features could be approximately regarded as IID-random, because the highest Pearson correlation coefficient reached only 0.482 while most of the Pearson correlation coefficients approximated zero. The Pearson correlation coefficient for 16-input features are much higher, as shown in Fig. 2b. The pseudo-IID random variables that we derived are acceptable for inverse-design-available deep-learning algorithms based on the probabilistic generative model.

## Supplementary information

Supplementary Information.

## Data Availability

All data generated or analyzed during this study are included in this published article (and its supplementary information file), and the datasets used for DNN and MVAE during the current study are available from the corresponding author on reasonable request.

## Code availability

The codes used for this study are available from the GitHub link at https://github.com/socoolblue/inverse-design-ML
