# Supplementary material for: Dirty engineering data-driven inverse prediction machine learning model
Source: Sci Rep. 2020 Nov 24;10:20443. doi: 10.1038/s41598-020-77575-0 (PMC7687896; doi:10.1038/s41598-020-77575-0)
Supplement: Supplementary file 1 — Supplementary Information. [file 41598_2020_77575_MOESM1_ESM.docx]

**Supplementary Information**

**Dirty engineering data-driven inverse prediction machine learning model**

Jin-Woong Lee,^†,1^ Woon Bae Park,^†,1^ Byung Do Lee,^1^ Seonghwan Kim,^2^ Nam Hoon Goo,^2,*^ and Kee-Sun Sohn^1,*^

†These authors contributed equally to this work

*Corresponding Authors: [kssohn@sejong.ac.kr](mailto:kssohn@sejong.ac.kr), [namhgoo@hyundai-steel.com](mailto:namhgoo@hyundai-steel.com)

^1^Faculty of Nanotechnology and Advanced Materials Engineering, Sejong University, Seoul 143-747, Republic of Korea

^2^Advanced Research Team, Hyundai Steel DangJin works, DangJin, Chungnam 31719, Republic of Korea

**Supplementary Discussion 1**

**Non-dominated sorting genetic algorithm (NSGA-II)**

Multi-objective optimization problems consist of multi-dimensional decision variable vectors and multi-dimensional fitness vectors. Consider a scenario where there are n decision variables and two fitness functions (the yield strength, *f_1_*(**x**), and the ultimate tensile strength, *f_2_*(**x**)). The n decision variables can be encoded into either n-dimensional decimal vectors or m-dimensional binary codes.

*f* : **R**^n^ 🡪 **R**^2^ or *f* : **B**^m^ 🡪 **R**^2^ (R: real number space, B: 0 or 1, m >> n)

Maximize *f_1_*(**x**) = y_1_, Minimize *f_2_*(**x**) = y_2_

Objective (fitness) function vector: **y** = (y_1_, y_2_)^T^ = ( f_1_(**x**), f_2_(**x**) )^T^ (1)

Decision variable: phosphor composition

Decimal vectors or binary code strings: **x** = (x_1_, x_2_, x_3_, • • • x_n_)^T^ or
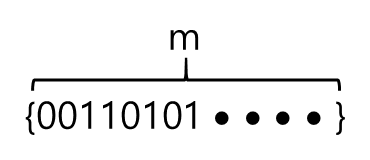


NSGA is based on a systematic classification of individuals, which is referred to as Pareto sorting. Before the selection process is performed (tournament selection was used in the present investigation), the population is ranked according to the Pareto dominance and all non-dominated individuals are classified into the first rank with a dummy fitness value. The individuals classified as non-dominated share a dummy fitness value, and are then removed from the population, whereupon a second layer of non-dominated individuals is then considered. This process continues with the remaining population until all individuals are classified. Since individuals in the first Pareto front have the maximum fitness value, they always receive more chances for selection than the rest of the population. This allows us to search for non-dominated solutions, to drive the first Pareto front toward a desirable direction (higher yield strength and ultimate tensile strength), and ultimately to reach a convergence.

Pareto optimality is the main issue for NSGA, which determines the relative dominance of **y*** and **y** (the thick letter denotes a vector). Each Pareto front represents a set of individuals (fitness function vectors) that are not strictly dominated by other individuals in the fitness function space. One fitness function vector, **y***, strictly dominates (or "is preferred to") other fitness function vectors, **y**, if each fitness of **y*** is not dominated by (≽) the corresponding fitness of **y** and at least one fitness of **y*** strictly dominates (≻) the corresponding fitness of **y**. This definition can be depicted as follows:

**y*** ≽ **y** if ∀i y_i_* ≽ y_i_ Λ ∃i y_i_* ≻ y_i_ (2)

The newly defined domination symbols, i.e., curved inequality symbols such as ≽ and ≻, should be differentiated from the conventional inequality symbols. In the present case, **y*** dominates **y** if { *f_1_*(**x***) ≧ *f_1_*(**x**) and *f_2_*(**x***) ≧ *f_2_*(**x**) } and { *f_1_*(**x***) > *f_1_*(**x**) or *f_2_*(x*****) > *f_2_*(**x**) }. The algorithm is similar to a simple GA, except for the classification of non-dominated fronts and the sharing operation. Fitness sharing, so-called niche sharing or niching, helps to distribute the population evenly over the Pareto front. In addition to niche sharing, either crowding-distance calculation or hyper-volume calculation can also be used for dummy fitness sharing. We adopted the crowding-distance calculation in the present investigation.

An elitism-involved NSGA, which is referred to as NSGA-II, was more recently developed to considerably reduce the complexity. In fact, we employed NSGA-II in the present study because it is known to lead to faster convergence. The NSGA-II process incorporates elitism in a unique manner, such that two subsequent generations are combined to find non-dominated solutions, which contrast with elitism operations for use in conventional GAs, wherein only a few elitist elements were simply selected out of the preceding generation.

**Supplementary Discussion 2**

**Conditional Variational Autoencoder (CVAE)**

CVAE is basically the same algorithm as VAE except for a slight alteration wherein the labeled X and Z data are used as an input for encoder and decoder, respectively. The label (Y) can be simply concatenated with either X or Z. A major issue is to assign reasonable labels to X and Z. The definition of the label matters in this case. In principle, the YS and UTS could be the label. If YS and UTS were simply adopted as labels, however, the continuous nature of the label would never reach a satisfactory training. Therefore, we refer to the Supplementary Discussion 3 “Transforming Regression to Classification”, wherein we simplified the YS and UTS data by categorizing them into three or five classes. We adopted the three-level-classification such that every X and Z entry was labeled with either 0 (high), 1 (intermediate), or 2 (low). Accordingly, we created three-dimensional one-hot vectors to be concatenated with X and Z.

The schematic representation for CVAE leading to a standard normal distribution, N(0, I), for the encoder output (Z) and a diagonal normal distribution, N(μ, σ^2^ᆞI), for the decoder output ($\hat{X}$) are shown in Figure S1. The Z data belonging to each of the three labels are represented in different colors, as shown in Figure S2 (a). Following the completion of the CVAE training, we pinpointed some data points belong to only the label ‘high’. The decoder output data corresponding to pinpointed Z data points along with the label ‘high’ are regarded as inverse-predicated solutions.

It is unfortunate, however, that the training result was not as good as the MVAE. This means that the overlap with the NSGA-II result was not as prominent as the MVAE case. So, we removed the CVAE result from the manuscript. More finely divided labels along with a greater amount of training data are required to improve the predictability for the CVAE approach.


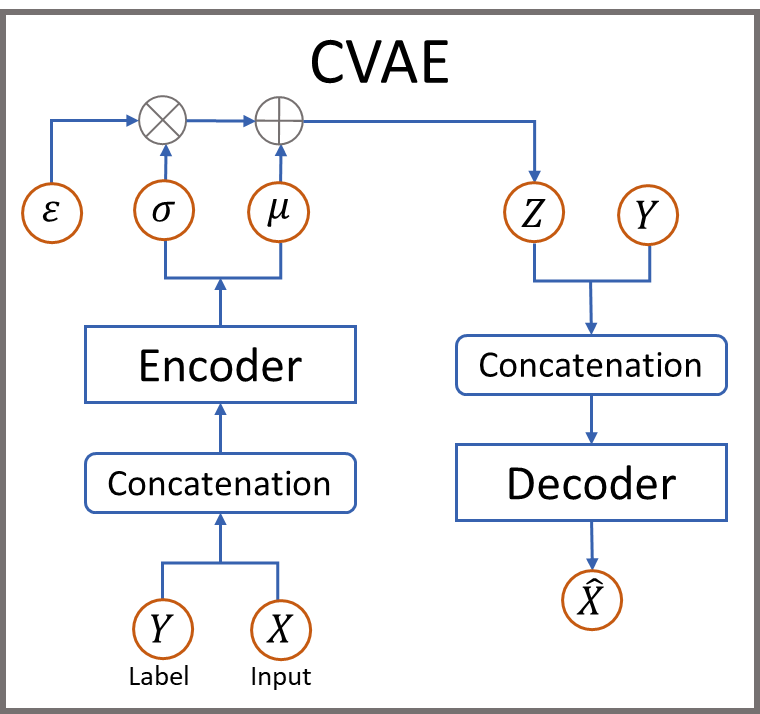


**Figure S1**. A schematic description of conditional variational auto-encoder (CVAE).

**Supplementary Discussion 3**

**Transforming Regression to Classification**

We simplified the output data consisting of two features (YS and UTS) by classifying the data into three or five classes, and then the regression problem could be transformed into a simple classification procedure. This drastic simplification never gave an accurate QCPR model, but it could be acceptable in the industry. In addition, this sort of simple compartmentalization of the output data could also be used as a conditional variational autoencoder (CVAE), which is discussed in the Supplementary information. The output data (YS and UTS) were divided by employing the well-known Pareto sorting method, and the boundary between the classes was determined by the Pareto ranks such that nearly equal populations can be assigned per each class. Figure S2 shows the class division results for three and five classes.

The ensuing DNN classification was implemented using the best architectures selected for both the 16- and 8-input-variable regression cases. The output layer consists of the same number of nodes as the number of classes, and cross entropy activation function was adopted. The test accuracy for the three-class-classification was 79.6 and 76.2% for the 16- and 8-input-feature DNNs, respectively. The five-class-classification result was dramatically deteriorated down to 58.9 and 58.2% for the 16- and 8-input-feature DNNs, respectively. Although this sort of rough prediction would be practical when a rough and facile judgment is required in a real-world industrial setting, the classification DNN model is not suitable for an inverse prediction.


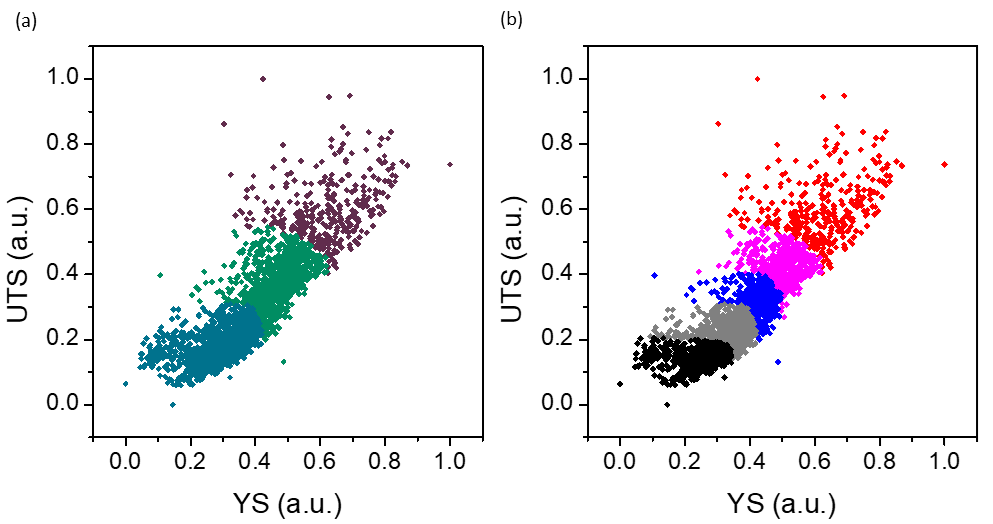


**Figure S2** The class division results for the output data (YS and UTS), which are divided into three and five classes.

**Table S1 (a).** 60 DNN architectures and the corresponding RMSEs for each of the 16-input-feature DNNs. The final selection was highlighted in bold font.

|  | Architecture | 5_Fold_Cross_Validation Cost | Test_Cost | Average |
| --- | --- | --- | --- | --- |
| layer4_1 | [16,4],[4,8],[8,4],[4,2] | [0.009066, 0.009206, 0.020419, 0.011526, 0.008568] | 0.009603 | 0.011398 |
| layer4_2 | [16,8],[8,16],[16,8],[8,2] | [0.008276, 0.007654, 0.009371, 0.009812, 0.009179] | 0.009066 | 0.008893 |
| layer4_3 | [16,16],[16,32],[32,16],[16,2] | [0.007739, 0.008201, 0.007943, 0.007728, 0.007831] | 0.006868 | 0.007719 |
| layer4_4 | [16,32],[32,64],[64,32],[32,2] | [0.008532, 0.008157, 0.007707, 0.007836, 0.008696] | 0.008678 | 0.008268 |
| layer4_5 | [16,64],[64,128],[128,64],[64,2] | [0.009174, 0.009653, 0.008509, 0.008164, 0.008967] | 0.009192 | 0.008943 |
| layer4_6 | [16,16],[16,8],[8,4],[4,2] | [0.009127, 0.008199, 0.008245, 0.021535, 0.008049] | 0.007203 | 0.010393 |
| layer4_7 | [16,32],[32,16],[16,8],[8,2] | [0.007807, 0.008053, 0.008425, 0.007954, 0.007193] | 0.008653 | 0.008014 |
| layer4_8 | **[16,64],[64,32],[32,16],[16,2]** | **[0.0072, 0.007545, 0.007701, 0.007351, 0.007961]** | **0.00832** | **0.00768** |
| layer4_9 | [16,128],[128,64],[64,32],[32,2] | [0.009334, 0.009652, 0.01009, 0.007669, 0.007363] | 0.007811 | 0.008653 |
| layer4_10 | [16,256],[256,128],[128,64],[64,2] | [0.009132, 0.009215, 0.009158, 0.009166, 0.008891] | 0.009534 | 0.009183 |
| layer4_11 | [16,4],[4,8],[8,16],[16,2] | [0.011198, 0.008712, 0.009536, 0.00904, 0.009675] | 0.008772 | 0.009489 |
| layer4_12 | [16,8],[8,16],[16,32],[32,2] | [0.007845, 0.007754, 0.008773, 0.008505, 0.008348] | 0.008587 | 0.008302 |
| layer4_13 | [16,16],[16,32],[32,64],[64,2] | [0.007573, 0.007909, 0.008615, 0.007906, 0.007526] | 0.006811 | 0.007723 |
| layer4_14 | [16,32],[32,64],[64,128],[128,2] | [0.008341, 0.008044, 0.009362, 0.008301, 0.008769] | 0.007707 | 0.008421 |
| layer4_15 | [16,64],[64,128],[128,256],[256,2] | [0.008701, 0.009471, 0.008907, 0.009125, 0.009503] | 0.009512 | 0.009203 |
| layer4_16 | [16,4],[4,16],[16,8],[8,2] | [0.011505, 0.010318, 0.008634, 0.008943, 0.009202] | 0.008193 | 0.009466 |
| layer4_17 | [16,8],[8,32],[32,16],[16,2] | [0.008083, 0.00782, 0.007715, 0.009761, 0.007977] | 0.008423 | 0.008296 |
| layer4_18 | [16,16],[16,64],[64,32],[32,2] | [0.008955, 0.007472, 0.007784, 0.008206, 0.007844] | 0.007848 | 0.008018 |
| layer4_19 | [16,32],[32,128],[128,64],[64,2] | [0.009485, 0.00938, 0.008162, 0.010037, 0.009464] | 0.007933 | 0.009077 |
| layer4_20 | [16,64],[64,256],[256,128],[128,2] | [0.010764, 0.010259, 0.010028, 0.007907, 0.009182] | 0.00956 | 0.009617 |
| layer6_1 | [16,4],[4,8],[8,16],[16,8],[8,4],[4,2] | [0.00898, 0.010181, 0.009331, 0.007651, 0.010667] | 0.009184 | 0.009332 |
| layer6_2 | [16,8],[8,16],[16,32],[32,16],[16,8],[8,2] | [0.009123, 0.008064, 0.007925, 0.008652, 0.007383] | 0.008077 | 0.008204 |
| layer6_3 | [16,16],[16,32],[32,64],[64,32],[32,16],[16,2] | [0.009212, 0.00929, 0.008157, 0.008584, 0.008355] | 0.007868 | 0.008578 |
| layer6_4 | [16,32],[32,64],[64,128],[128,64],[64,32],[32,2] | [0.007959, 0.010103, 0.009856, 0.008988, 0.008937] | 0.010031 | 0.009312 |
| layer6_5 | [16,64],[64,128],[128,256],[256,128],[128,64],[64,2] | [0.009663, 0.010924, 0.008913, 0.009127, 0.010742] | 0.008953 | 0.00972 |
| layer6_6 | [16,64],[64,32],[32,16],[16,8],[8,4],[4,2] | [0.007314, 0.021517, 0.008473, 0.007307, 0.009249] | 0.009294 | 0.010526 |
| layer6_7 | [16,128],[128,64],[64,32],[32,16],[16,8],[8,2] | [0.008814, 0.008961, 0.008673, 0.009507, 0.008392] | 0.009018 | 0.008894 |
| layer6_8 | [16,256],[256,128],[128,64],[64,32],[32,16],[16,2] | [0.008827, 0.009533, 0.009226, 0.010632, 0.009402] | 0.008957 | 0.00943 |
| layer6_9 | [16,512],[512,256],[256,128],[128,64],[64,32],[32,2] | [0.010072, 0.008557, 0.008736, 0.009329, 0.008777] | 0.009351 | 0.009137 |
| layer6_10 | [16,1024],[1024,512],[512,256],[256,128],[128,64],[64,2] | [0.009324, 0.00866, 0.009032, 0.009005, 0.007246] | 0.00801 | 0.008546 |
| layer6_11 | [16,4],[4,8],[8,16],[16,32],[32,64],[64,2] | [0.007631, 0.009242, 0.008647, 0.008267, 0.010481] | 0.012164 | 0.009405 |
| layer6_12 | [16,8],[8,16],[16,32],[32,64],[64,128],[128,2] | [0.008486, 0.007878, 0.009022, 0.009186, 0.008305] | 0.008186 | 0.00851 |
| layer6_13 | [16,16],[16,32],[32,64],[64,128],[128,256],[256,2] | [0.008537, 0.008696, 0.009124, 0.007974, 0.009719] | 0.009763 | 0.008969 |
| layer6_14 | [16,32],[32,64],[64,128],[128,256],[256,512],[512,2] | [0.009391, 0.009071, 0.008993, 0.009042, 0.00868] | 0.008949 | 0.009021 |
| layer6_15 | [16,64],[64,128],[128,256],[256,512],[512,1024],[1024,2] | [0.008358, 0.009057, 0.008448, 0.021055, 0.008519] | 0.007536 | 0.010495 |
| layer6_16 | [16,4],[4,16],[16,8],[8,32],[32,16],[16,2] | [0.008457, 0.008274, 0.009415, 0.008767, 0.010496] | 0.010595 | 0.009334 |
| layer6_17 | [16,8],[8,32],[32,16],[16,64],[64,32],[32,2] | [0.008578, 0.008347, 0.008208, 0.008736, 0.008] | 0.008974 | 0.008474 |
| layer6_18 | [16,16],[16,64],[64,32],[32,128],[128,64],[64,2] | [0.008839, 0.009703, 0.008104, 0.007926, 0.007845] | 0.008248 | 0.008444 |
| layer6_19 | [16,32],[32,128],[128,64],[64,256],[256,128],[128,2] | [0.00756, 0.007857, 0.008126, 0.00874, 0.009177] | 0.008771 | 0.008372 |
| layer6_20 | [16,64],[64,256],[256,128],[128,512],[512,256],[256,2] | [0.008606, 0.006966, 0.010236, 0.010479, 0.008039] | 0.009453 | 0.008963 |
| layer8_1 | [16,4],[4,8],[8,16],[16,32],[32,16],[16,8],[8,4],[4,2] | [0.00804, 0.020791, 0.010705, 0.021271, 0.011092] | 0.01159 | 0.013915 |
| layer8_2 | [16,8],[8,16],[16,32],[32,64],[64,32],[32,16],[16,8],[8,2] | [0.011436, 0.007509, 0.008612, 0.008587, 0.008042] | 0.008384 | 0.008762 |
| layer8_3 | [16,16],[16,32],[32,64],[64,128],[128,64],[64,32],[32,16],[16,2] | [0.008754, 0.008529, 0.008921, 0.009229, 0.009701] | 0.008392 | 0.008921 |
| layer8_4 | [16,32],[32,64],[64,128],[128,256],[256,128],[128,64],[64,32],[32,2] | [0.008326, 0.008322, 0.008893, 0.008295, 0.008331] | 0.009373 | 0.00859 |
| layer8_5 | [16,64],[64,128],[128,256],[256,512],[512,256],[256,128],[128,64],[64,2] | [0.009195, 0.008892, 0.007661, 0.009658, 0.009739] | 0.007811 | 0.008826 |
| layer8_6 | [16,256],[256,128],[128,64],[64,32],[32,16],[16,8],[8,4],[4,2] | [0.008793, 0.0218, 0.021722, 0.021395, 0.009837] | 0.008178 | 0.015288 |
| layer8_7 | [16,512],[512,256],[256,128],[128,64],[64,32],[32,16],[16,8],[8,2] | [0.010003, 0.008992, 0.009676, 0.009413, 0.010128] | 0.007727 | 0.009323 |
| layer8_8 | [16,1024],[1024,512],[512,256],[256,128],[128,64],[64,32],[32,16],[16,2] | [0.007965, 0.009164, 0.009392, 0.008337, 0.00989] | 0.008866 | 0.008936 |
| layer8_9 | [16,2048],[2048,1024],[1024,512],[512,256],[256,128],[128,64],[64,32],[32,2] | [0.008441, 0.00873, 0.02057, 0.009233, 0.007453] | 0.008843 | 0.010545 |
| layer8_10 | [16,4096],[4096,2048],[2048,1024],[1024,512],[512,256],[256,128],[128,64],[64,2] | [0.023664, 0.020436, 0.020813, 0.02072, 0.020991] | 0.021548 | 0.021362 |
| layer8_11 | [16,4],[4,8],[8,16],[16,32],[32,64],[64,128],[128,256],[256,2] | [0.008217, 0.021754, 0.010297, 0.00868, 0.010775] | 0.011568 | 0.011882 |
| layer8_12 | [16,8],[8,16],[16,32],[32,64],[64,128],[128,256],[256,512],[512,2] | [0.012173, 0.009465, 0.01012, 0.007758, 0.008503] | 0.007197 | 0.009203 |
| layer8_13 | [16,16],[16,32],[32,64],[64,128],[128,256],[256,512],[512,1024],[1024,2] | [0.008444, 0.008482, 0.008333, 0.010669, 0.008959] | 0.008094 | 0.00883 |
| layer8_14 | [16,32],[32,64],[64,128],[128,256],[256,512],[512,1024],[1024,2048],[2048,2] | [0.011273, 0.011621, 0.011163, 0.0101, 0.007569] | 0.006773 | 0.00975 |
| layer8_15 | [16,64],[64,128],[128,256],[256,512],[512,1024],[1024,2048],[2048,4096],[4096,2] | [0.057049, 0.015256, 0.01442, 0.012174, 0.02194] | 0.022893 | 0.023955 |
| layer8_16 | [16,4],[4,16],[16,8],[8,32],[32,16],[16,64],[64,32],[32,2] | [0.021664, 0.020943, 0.009266, 0.0086, 0.012206] | 0.01158 | 0.014043 |
| layer8_17 | [16,8],[8,32],[32,16],[16,64],[64,32],[32,128],[128,64],[64,2] | [0.010737, 0.007375, 0.008036, 0.007636, 0.009137] | 0.008847 | 0.008628 |
| layer8_18 | [16,16],[16,64],[64,32],[32,128],[128,64],[64,256],[256,128],[128,2] | [0.010147, 0.008398, 0.00879, 0.008279, 0.007786] | 0.007391 | 0.008465 |
| layer8_19 | [16,32],[32,128],[128,64],[64,256],[256,128],[128,512],[512,256],[256,2] | [0.007802, 0.008369, 0.009193, 0.008149, 0.008704] | 0.010152 | 0.008728 |
| layer8_20 | [16,64],[64,256],[256,128],[128,512],[512,256],[256,1024],[1024,512],[512,2] | [0.022497, 0.020518, 0.020705, 0.019852, 0.008115] | 0.007306 | 0.016499 |

**Table S1 (b)**. 60 DNN architectures and the corresponding RMSEs for each of the 8-input-feature DNNs. The final selection was highlighted in bold font.

|  | Architecture | 5_Fold_Cross_Validation Cost | Test_Cost | Average |
| --- | --- | --- | --- | --- |
| layer4_1 | [8,4],[4,8],[8,4],[4,2] | [0.010621, 0.012323, 0.02069, 0.011179, 0.010508] | 0.010729 | 0.012675 |
| layer4_2 | [8,8],[8,16],[16,8],[8,2] | [0.010109, 0.010024, 0.01094, 0.011053, 0.010818] | 0.01069 | 0.010606 |
| layer4_3 | [8,16],[16,32],[32,16],[16,2] | [0.009358, 0.008446, 0.00898, 0.0099, 0.009751] | 0.00913 | 0.009261 |
| layer4_4 | **[8,32],[32,64],[64,32],[32,2]** | **[0.009032, 0.007686, 0.00796, 0.008628, 0.007704]** | **0.008237** | **0.00821** |
| layer4_5 | [8,64],[64,128],[128,64],[64,2] | [0.009631, 0.007606, 0.008573, 0.007199, 0.008783] | 0.009405 | 0.008533 |
| layer4_6 | [8,16],[16,8],[8,4],[4,2] | [0.010737, 0.010436, 0.010223, 0.020764, 0.009759] | 0.010367 | 0.012048 |
| layer4_7 | [8,32],[32,16],[16,8],[8,2] | [0.009156, 0.00955, 0.010004, 0.009355, 0.010203] | 0.010296 | 0.009761 |
| layer4_8 | [8,64],[64,32],[32,16],[16,2] | [0.00824, 0.00827, 0.007331, 0.009687, 0.008558] | 0.008654 | 0.008457 |
| layer4_9 | [8,128],[128,64],[64,32],[32,2] | [0.008014, 0.0085, 0.009565, 0.007955, 0.008896] | 0.008948 | 0.008646 |
| layer4_10 | [8,256],[256,128],[128,64],[64,2] | [0.008655, 0.009321, 0.007645, 0.008494, 0.007822] | 0.008129 | 0.008344 |
| layer4_11 | [8,4],[4,8],[8,16],[16,2] | [0.011386, 0.01226, 0.010049, 0.011325, 0.011871] | 0.010867 | 0.011293 |
| layer4_12 | [8,8],[8,16],[16,32],[32,2] | [0.00974, 0.010197, 0.009353, 0.010456, 0.00926] | 0.011559 | 0.010094 |
| layer4_13 | [8,16],[16,32],[32,64],[64,2] | [0.010002, 0.009466, 0.008403, 0.008717, 0.008447] | 0.008418 | 0.008909 |
| layer4_14 | [8,32],[32,64],[64,128],[128,2] | [0.008397, 0.008532, 0.009294, 0.009203, 0.009003] | 0.007992 | 0.008737 |
| layer4_15 | [8,64],[64,128],[128,256],[256,2] | [0.00795, 0.010092, 0.009747, 0.008764, 0.009576] | 0.009083 | 0.009202 |
| layer4_16 | [8,4],[4,16],[16,8],[8,2] | [0.011087, 0.011133, 0.012006, 0.01024, 0.011345] | 0.010868 | 0.011113 |
| layer4_17 | [8,8],[8,32],[32,16],[16,2] | [0.010816, 0.009012, 0.010171, 0.010332, 0.009951] | 0.009941 | 0.010037 |
| layer4_18 | [8,16],[16,64],[64,32],[32,2] | [0.008012, 0.00793, 0.008841, 0.008122, 0.008989] | 0.00847 | 0.008394 |
| layer4_19 | [8,32],[32,128],[128,64],[64,2] | [0.012997, 0.008424, 0.009547, 0.008312, 0.009714] | 0.008506 | 0.009583 |
| layer4_20 | [8,64],[64,256],[256,128],[128,2] | [0.008681, 0.010702, 0.009056, 0.00857, 0.010063] | 0.009867 | 0.00949 |
| layer6_1 | [8,4],[4,8],[8,16],[16,8],[8,4],[4,2] | [0.010748, 0.020895, 0.011074, 0.009968, 0.011113] | 0.011238 | 0.012506 |
| layer6_2 | [8,8],[8,16],[16,32],[32,16],[16,8],[8,2] | [0.008915, 0.010432, 0.009977, 0.009862, 0.008141] | 0.008448 | 0.009296 |
| layer6_3 | [8,16],[16,32],[32,64],[64,32],[32,16],[16,2] | [0.008814, 0.008348, 0.008442, 0.008334, 0.008578] | 0.009182 | 0.008617 |
| layer6_4 | [8,32],[32,64],[64,128],[128,64],[64,32],[32,2] | [0.009582, 0.00865, 0.009688, 0.01014, 0.010092] | 0.01022 | 0.009729 |
| layer6_5 | [8,64],[64,128],[128,256],[256,128],[128,64],[64,2] | [0.010187, 0.009356, 0.009572, 0.008114, 0.009251] | 0.009117 | 0.009266 |
| layer6_6 | [8,64],[64,32],[32,16],[16,8],[8,4],[4,2] | [0.008608, 0.020433, 0.008351, 0.022772, 0.008914] | 0.009121 | 0.013033 |
| layer6_7 | [8,128],[128,64],[64,32],[32,16],[16,8],[8,2] | [0.008151, 0.008561, 0.008765, 0.010173, 0.0076] | 0.00943 | 0.00878 |
| layer6_8 | [8,256],[256,128],[128,64],[64,32],[32,16],[16,2] | [0.010491, 0.010126, 0.009578, 0.009771, 0.00968] | 0.009081 | 0.009788 |
| layer6_9 | [8,512],[512,256],[256,128],[128,64],[64,32],[32,2] | [0.01068, 0.008422, 0.009988, 0.009164, 0.010321] | 0.009459 | 0.009672 |
| layer6_10 | [8,1024],[1024,512],[512,256],[256,128],[128,64],[64,2] | [0.008285, 0.00829, 0.00911, 0.008287, 0.0085] | 0.008723 | 0.008533 |
| layer6_11 | [8,4],[4,8],[8,16],[16,32],[32,64],[64,2] | [0.009107, 0.012469, 0.008373, 0.010547, 0.010256] | 0.010186 | 0.010156 |
| layer6_12 | [8,8],[8,16],[16,32],[32,64],[64,128],[128,2] | [0.010724, 0.00972, 0.009279, 0.007988, 0.011805] | 0.009821 | 0.00989 |
| layer6_13 | [8,16],[16,32],[32,64],[64,128],[128,256],[256,2] | [0.009015, 0.009762, 0.00921, 0.008773, 0.008323] | 0.008329 | 0.008902 |
| layer6_14 | [8,32],[32,64],[64,128],[128,256],[256,512],[512,2] | [0.010198, 0.009048, 0.009491, 0.010259, 0.009099] | 0.009473 | 0.009594 |
| layer6_15 | [8,64],[64,128],[128,256],[256,512],[512,1024],[1024,2] | [0.009278, 0.008605, 0.009506, 0.00896, 0.007516] | 0.007676 | 0.00859 |
| layer6_16 | [8,4],[4,16],[16,8],[8,32],[32,16],[16,2] | [0.008784, 0.010228, 0.009642, 0.010216, 0.010158] | 0.011368 | 0.010066 |
| layer6_17 | [8,8],[8,32],[32,16],[16,64],[64,32],[32,2] | [0.008152, 0.008182, 0.008998, 0.009635, 0.008122] | 0.007252 | 0.00839 |
| layer6_18 | [8,16],[16,64],[64,32],[32,128],[128,64],[64,2] | [0.008826, 0.008797, 0.008815, 0.00758, 0.009138] | 0.009627 | 0.008797 |
| layer6_19 | [8,32],[32,128],[128,64],[64,256],[256,128],[128,2] | [0.011349, 0.021206, 0.008432, 0.008672, 0.008968] | 0.009238 | 0.011311 |
| layer6_20 | [8,64],[64,256],[256,128],[128,512],[512,256],[256,2] | [0.007796, 0.009189, 0.009506, 0.010311, 0.008992] | 0.008665 | 0.009076 |
| layer8_1 | [8,4],[4,8],[8,16],[16,32],[32,16],[16,8],[8,4],[4,2] | [0.011908, 0.019804, 0.011762, 0.021445, 0.010117] | 0.011438 | 0.014412 |
| layer8_2 | [8,8],[8,16],[16,32],[32,64],[64,32],[32,16],[16,8],[8,2] | [0.009486, 0.008688, 0.008157, 0.010672, 0.008595] | 0.008916 | 0.009086 |
| layer8_3 | [8,16],[16,32],[32,64],[64,128],[128,64],[64,32],[32,16],[16,2] | [0.009602, 0.00973, 0.008474, 0.009436, 0.009177] | 0.008948 | 0.009228 |
| layer8_4 | [8,32],[32,64],[64,128],[128,256],[256,128],[128,64],[64,32],[32,2] | [0.008893, 0.009246, 0.007433, 0.009427, 0.010938] | 0.01092 | 0.009476 |
| layer8_5 | [8,64],[64,128],[128,256],[256,512],[512,256],[256,128],[128,64],[64,2] | [0.010661, 0.007737, 0.010369, 0.009276, 0.00994] | 0.009202 | 0.009531 |
| layer8_6 | [8,256],[256,128],[128,64],[64,32],[32,16],[16,8],[8,4],[4,2] | [0.02209, 0.022416, 0.021078, 0.02058, 0.009785] | 0.009417 | 0.017561 |
| layer8_7 | [8,512],[512,256],[256,128],[128,64],[64,32],[32,16],[16,8],[8,2] | [0.009051, 0.009036, 0.00827, 0.010132, 0.01034] | 0.009907 | 0.009456 |
| layer8_8 | [8,1024],[1024,512],[512,256],[256,128],[128,64],[64,32],[32,16],[16,2] | [0.008531, 0.009245, 0.008005, 0.008424, 0.008525] | 0.007877 | 0.008434 |
| layer8_9 | [8,2048],[2048,1024],[1024,512],[512,256],[256,128],[128,64],[64,32],[32,2] | [0.021594, 0.008838, 0.02137, 0.009778, 0.008238] | 0.00882 | 0.013106 |
| layer8_10 | [8,4096],[4096,2048],[2048,1024],[1024,512],[512,256],[256,128],[128,64],[64,2] | [0.023482, 0.01013, 0.020639, 0.019586, 0.021344] | 0.022375 | 0.019593 |
| layer8_11 | [8,4],[4,8],[8,16],[16,32],[32,64],[64,128],[128,256],[256,2] | [0.008087, 0.009646, 0.012348, 0.008434, 0.010574] | 0.010407 | 0.009916 |
| layer8_12 | [8,8],[8,16],[16,32],[32,64],[64,128],[128,256],[256,512],[512,2] | [0.010711, 0.009996, 0.00894, 0.011014, 0.009295] | 0.007479 | 0.009573 |
| layer8_13 | [8,16],[16,32],[32,64],[64,128],[128,256],[256,512],[512,1024],[1024,2] | [0.008082, 0.008954, 0.010738, 0.009271, 0.010029] | 0.010747 | 0.009637 |
| layer8_14 | [8,32],[32,64],[64,128],[128,256],[256,512],[512,1024],[1024,2048],[2048,2] | [0.009103, 0.018944, 0.015518, 0.022023, 0.011314] | 0.011964 | 0.014811 |
| layer8_15 | [8,64],[64,128],[128,256],[256,512],[512,1024],[1024,2048],[2048,4096],[4096,2] | [0.012706, 0.021498, 0.021122, 0.014321, 0.011368] | 0.011949 | 0.015494 |
| layer8_16 | [8,4],[4,16],[16,8],[8,32],[32,16],[16,64],[64,32],[32,2] | [0.009577, 0.011076, 0.012027, 0.009315, 0.01039] | 0.010777 | 0.010527 |
| layer8_17 | [8,8],[8,32],[32,16],[16,64],[64,32],[32,128],[128,64],[64,2] | [0.010529, 0.008933, 0.010161, 0.008664, 0.009084] | 0.010573 | 0.009657 |
| layer8_18 | [8,16],[16,64],[64,32],[32,128],[128,64],[64,256],[256,128],[128,2] | [0.009451, 0.00922, 0.008739, 0.010512, 0.008556] | 0.009185 | 0.009277 |
| layer8_19 | [8,32],[32,128],[128,64],[64,256],[256,128],[128,512],[512,256],[256,2] | [0.008985, 0.008942, 0.009342, 0.009233, 0.008995] | 0.007958 | 0.008909 |
| layer8_20 | [8,64],[64,256],[256,128],[128,512],[512,256],[256,1024],[1024,512],[512,2] | [0.020883, 0.021033, 0.022405, 0.022336, 0.011206] | 0.012652 | 0.018419 |

**Table S2**. The MSE and R^2^ for the regression and the test accuracy for classification for KNN, SVM, RF, and GPR for comparison with the DNN.

|  | KNN (16_input) | KNN (8_input) | RF (16_input) | RF (8_input) | SVM (16_input) | SVM (8_input) | DNN (16_input) | DNN (8_input) | GP  (16_input) | GP  (8_input) |
| --- | --- | --- | --- | --- | --- | --- | --- | --- | --- | --- |
| Regression (R^2^_score) | 0.659 | 0.6816 | 0.6638 | 0.6791 | 0.6197 | 0.6388 | 0.6102 | 0.6026 | 0.6205 | 0.6618 |
| Regression (MSE) | 0.0076 | 0.0071 | 0.0075 | 0.0072 | 0.0085 | 0.0081 | 0.0087 | 0.0089 | 0.0085 | 0.0075 |
| 3_class_ Classification (Accuracy) | 0.7883 | 0.7883 | 0.8099 | 0.8232 | 0.7677 | 0.7677 | 0.7955 | 0.7616 | 0.7626 | 0.7246 |
| 5_class_ Classification (Accuracy) | 0.5683 | 0.5714 | 0.6403 | 0.629 | 0.5406 | 0.5519 | 0.5889 | 0.5817 | 0.5365 | 0.4666 |

**Table S3**. The final pinpointed solutions from both the NSGA-II and MVAE inverse prediction strategies. The so-called ‘pseudo-overlap’ method was used for the final selection of the solutions. The solutions belonging to the first three Pareto frontiers shown in Figure 4 are highlighted in bold font.

| C | Si | P | S | Ti | Time | Temperature | Mn | Cu | Sn | Ni | Cr | Mo | V | Nb | Ca | Class |
| --- | --- | --- | --- | --- | --- | --- | --- | --- | --- | --- | --- | --- | --- | --- | --- | --- |
| 0.069 | 0.225 | 0.006 | 0.002 | 0.014 | 301 | 1173 | 1.182 | 0.146 | 0.001 | 0.142 | 0.127 | 0 | 0.023 | 0.044 | 0.002 | NSGA-II |
| 0.09 | 0.295 | 0.01 | 0.003 | 0.014 | 349 | 1088 | 1.182 | 0.146 | 0.003 | 0.142 | 0.127 | 0 | 0.023 | 0.044 | 0.002 | MVAE |
| 0.082 | **0.285** | **0.006** | **0.004** | **0.016** | **489** | **1162** | **1.182** | **0.146** | **0.001** | **0.142** | **0.127** | **0** | **0.023** | **0.044** | **0.002** | **NSGA-II** |
| 0.089 | 0.312 | 0.009 | 0.002 | 0.014 | 328 | 1082 | 1.182 | 0.146 | 0.003 | 0.142 | 0.15 | 0 | 0.023 | 0.044 | 0.002 | MVAE |
| 0.08 | 0.255 | 0.005 | 0.002 | 0.013 | 255 | 1023 | 1.46 | 0.031 | 0.002 | 0.016 | 0.023 | 0 | 0.001 | 0.034 | 0.001 | MVAE |
| 0.081 | 0.245 | 0.014 | 0.003 | 0.015 | 254 | 1201 | 1.182 | 0.146 | 0.003 | 0.142 | 0.127 | 0.01 | 0.023 | 0.044 | 0.002 | MVAE |
| 0.092 | 0.303 | 0.01 | 0.003 | 0.014 | 344 | 1094 | 1.182 | 0.146 | 0.003 | 0.142 | 0.127 | 0 | 0.023 | 0.044 | 0.002 | MVAE |
| 0.061 | 0.313 | 0.015 | 0.002 | 0.011 | 425 | 1012 | 1.182 | 0.146 | 0.001 | 0.142 | 0.127 | 0 | 0.023 | 0.044 | 0.002 | NSGA-II |
| 0.076 | 0.223 | 0.011 | 0.002 | 0.014 | 206 | 1053 | 1.182 | 0.146 | 0.001 | 0.142 | 0.127 | 0 | 0.023 | 0.044 | 0.002 | NSGA-II |
| 0.057 | 0.243 | 0.015 | 0.003 | 0.014 | 265 | 1206 | 1.182 | 0.146 | 0.002 | 0.142 | 0.15 | 0 | 0.023 | 0.044 | 0.002 | MVAE |
| 0.092 | 0.296 | 0.01 | 0.003 | 0.014 | 349 | 1116 | 1.182 | 0.146 | 0.002 | 0.142 | 0.127 | 0 | 0.023 | 0.044 | 0.002 | MVAE |
| 0.084 | 0.228 | 0.007 | 0.003 | 0.012 | 286 | 1165 | 1.255 | 0.157 | 0.001 | 0.016 | 0.054 | 0.006 | 0.005 | 0.026 | 0.002 | NSGA-II |
| 0.091 | 0.28 | 0.012 | 0.003 | 0.014 | 366 | 1138 | 1.182 | 0.146 | 0.001 | 0.142 | 0.15 | 0 | 0.023 | 0.044 | 0.002 | MVAE |
| 0.082 | 0.319 | 0.007 | 0.002 | 0.013 | 301 | 1052 | 1.182 | 0.146 | 0.003 | 0.142 | 0.127 | 0.01 | 0.023 | 0.044 | 0.002 | MVAE |
| 0.057 | 0.235 | 0.009 | 0.001 | 0.011 | 387 | 969 | 1.46 | 0.146 | 0.002 | 0.189 | 0.15 | 0 | 0.023 | 0.044 | 0.002 | NSGA-II |
| 0.074 | 0.297 | 0.008 | 0.001 | 0.01 | 173 | 1218 | 1.182 | 0.146 | 0.003 | 0.142 | 0.15 | 0 | 0.023 | 0.044 | 0.002 | MVAE |
| 0.058 | 0.26 | 0.005 | 0.004 | 0.014 | 312 | 1048 | 1.46 | 0.146 | 0.002 | 0.189 | 0.15 | 0 | 0.023 | 0.044 | 0.002 | NSGA-II |
| 0.088 | 0.304 | 0.009 | 0.003 | 0.014 | 334 | 1097 | 1.182 | 0.146 | 0.003 | 0.142 | 0.127 | 0 | 0.023 | 0.044 | 0.002 | MVAE |
| 0.09 | 0.311 | 0.009 | 0.002 | 0.014 | 330 | 1086 | 1.182 | 0.146 | 0.002 | 0.142 | 0.15 | 0 | 0.023 | 0.044 | 0.002 | MVAE |
| 0.055 | 0.248 | 0.008 | 0.001 | 0.011 | 495 | 939 | 1.182 | 0.146 | 0.001 | 0.142 | 0.127 | 0 | 0.023 | 0.044 | 0.002 | NSGA-II |
| 0.088 | 0.317 | 0.008 | 0.002 | 0.013 | 319 | 1066 | 1.182 | 0.146 | 0.003 | 0.142 | 0.15 | 0 | 0.023 | 0.044 | 0.002 | MVAE |
| 0.082 | 0.317 | 0.007 | 0.002 | 0.013 | 305 | 1051 | 1.182 | 0.146 | 0.003 | 0.142 | 0.127 | 0 | 0.023 | 0.044 | 0.002 | MVAE |
| 0.093 | 0.301 | 0.01 | 0.003 | 0.014 | 350 | 1106 | 1.182 | 0.146 | 0.003 | 0.142 | 0.127 | 0 | 0.023 | 0.044 | 0.002 | MVAE |
| 0.082 | 0.321 | 0.007 | 0.002 | 0.013 | 300 | 1039 | 1.182 | 0.146 | 0.003 | 0.142 | 0.15 | 0 | 0.023 | 0.044 | 0.002 | MVAE |
| 0.07 | 0.313 | 0.015 | 0.002 | 0.011 | 425 | 1012 | 1.182 | 0.146 | 0.001 | 0.142 | 0.127 | 0 | 0.023 | 0.044 | 0.002 | NSGA-II |
| 0.078 | 0.253 | 0.011 | 0.005 | 0.01 | 258 | 1193 | 1.182 | 0.146 | 0.003 | 0.142 | 0.127 | 0.01 | 0.023 | 0.044 | 0.002 | MVAE |
| 0.081 | 0.24 | 0.01 | 0.003 | 0.013 | 255 | 1171 | 1.182 | 0.146 | 0.001 | 0.142 | 0.127 | 0 | 0.023 | 0.044 | 0.002 | NSGA-II |
| 0.055 | 0.239 | 0.013 | 0.002 | 0.011 | 195 | 1112 | 1.185 | 0.032 | 0.002 | 0.14 | 0.112 | 0.001 | 0.016 | 0.029 | 0.001 | NSGA-II |
| 0.083 | 0.317 | 0.007 | 0.002 | 0.013 | 310 | 1041 | 1.182 | 0.146 | 0.003 | 0.142 | 0.127 | 0 | 0.023 | 0.044 | 0.002 | MVAE |
| 0.079 | 0.255 | 0.005 | 0.002 | 0.013 | 256 | 1009 | 1.46 | 0.031 | 0.002 | 0.016 | 0.023 | 0 | 0.001 | 0.034 | 0.001 | MVAE |
| 0.091 | 0.293 | 0.011 | 0.003 | 0.014 | 359 | 1138 | 1.182 | 0.146 | 0.002 | 0.142 | 0.127 | 0 | 0.023 | 0.044 | 0.002 | MVAE |
| 0.08 | 0.221 | 0.012 | 0.002 | 0.015 | 316 | 1162 | 1.182 | 0.146 | 0.001 | 0.142 | 0.127 | 0 | 0.023 | 0.044 | 0.002 | NSGA-II |
| 0.054 | 0.24 | 0.013 | 0.002 | 0.015 | 310 | 1178 | 1.182 | 0.146 | 0.002 | 0.142 | 0.15 | 0 | 0.023 | 0.044 | 0.002 | MVAE |
| 0.089 | 0.302 | 0.01 | 0.002 | 0.014 | 327 | 1094 | 1.182 | 0.146 | 0.002 | 0.142 | 0.15 | 0 | 0.023 | 0.044 | 0.002 | MVAE |
| 0.086 | 0.314 | 0.008 | 0.002 | 0.013 | 321 | 1082 | 1.182 | 0.146 | 0.003 | 0.142 | 0.15 | 0 | 0.023 | 0.044 | 0.002 | MVAE |
| 0.064 | 0.23 | 0.015 | 0.003 | 0.01 | 383 | 977 | 1.182 | 0.146 | 0.001 | 0.142 | 0.127 | 0 | 0.023 | 0.044 | 0.002 | NSGA-II |
| 0.061 | 0.253 | 0.013 | 0.003 | 0.011 | 425 | 1148 | 1.182 | 0.146 | 0.001 | 0.142 | 0.127 | 0 | 0.023 | 0.044 | 0.002 | NSGA-II |
| 0.073 | 0.248 | 0.008 | 0.004 | 0.015 | 393 | 1076 | 1.46 | 0.146 | 0.002 | 0.189 | 0.15 | 0 | 0.023 | 0.044 | 0.002 | NSGA-II |
| 0.059 | 0.222 | 0.006 | 0.003 | 0.015 | 338 | 1014 | 1.46 | 0.146 | 0.002 | 0.189 | 0.15 | 0 | 0.023 | 0.044 | 0.002 | NSGA-II |
| 0.058 | 0.236 | 0.017 | 0.003 | 0.013 | 321 | 1032 | 1.182 | 0.146 | 0.002 | 0.142 | 0.15 | 0 | 0.023 | 0.044 | 0.002 | MVAE |
| 0.082 | 0.243 | 0.007 | 0.002 | 0.014 | 188 | 1117 | 1.182 | 0.146 | 0.001 | 0.142 | 0.127 | 0 | 0.023 | 0.044 | 0.002 | NSGA-II |
| 0.054 | 0.223 | 0.01 | 0.002 | 0.015 | 269 | 1039 | 1.46 | 0.146 | 0.002 | 0.189 | 0.127 | 0 | 0.023 | 0.044 | 0.002 | MVAE |
| 0.074 | 0.22 | 0.007 | 0.002 | 0.015 | 330 | 967 | 1.46 | 0.146 | 0.002 | 0.189 | 0.15 | 0 | 0.023 | 0.044 | 0.002 | NSGA-II |
| 0.09 | 0.307 | 0.009 | 0.002 | 0.013 | 334 | 1091 | 1.182 | 0.146 | 0.003 | 0.142 | 0.127 | 0 | 0.023 | 0.044 | 0.002 | MVAE |
| 0.076 | 0.221 | 0.008 | 0.001 | 0.014 | 288 | 1199 | 1.46 | 0.146 | 0.002 | 0.189 | 0.15 | 0 | 0.023 | 0.044 | 0.002 | NSGA-II |
| 0.084 | 0.226 | 0.007 | 0.003 | 0.013 | 354 | 1263 | 1.182 | 0.146 | 0.001 | 0.142 | 0.127 | 0 | 0.023 | 0.044 | 0.002 | NSGA-II |
| 0.076 | 0.226 | 0.009 | 0.001 | 0.015 | 205 | 1156 | 1.46 | 0.146 | 0.002 | 0.189 | 0.15 | 0 | 0.023 | 0.044 | 0.002 | NSGA-II |
| 0.081 | 0.316 | 0.007 | 0.002 | 0.014 | 302 | 1038 | 1.182 | 0.146 | 0.003 | 0.142 | 0.127 | 0.01 | 0.023 | 0.044 | 0.002 | MVAE |
| 0.074 | 0.231 | 0.01 | 0.001 | 0.014 | 266 | 974 | 1.46 | 0.146 | 0.002 | 0.189 | 0.15 | 0 | 0.023 | 0.044 | 0.002 | NSGA-II |
| 0.091 | 0.299 | 0.01 | 0.003 | 0.013 | 345 | 1111 | 1.182 | 0.146 | 0.002 | 0.142 | 0.14 | 0 | 0.023 | 0.044 | 0.002 | MVAE |
| 0.056 | 0.267 | 0.008 | 0.002 | 0.013 | 434 | 963 | 1.207 | 0.156 | 0.002 | 0.076 | 0.031 | 0.001 | 0.015 | 0.039 | 0.002 | NSGA-II |
| 0.092 | 0.294 | 0.011 | 0.003 | 0.014 | 358 | 1126 | 1.182 | 0.146 | 0.002 | 0.142 | 0.15 | 0 | 0.023 | 0.044 | 0.002 | MVAE |
| 0.092 | 0.288 | 0.011 | 0.003 | 0.014 | 363 | 1122 | 1.182 | 0.146 | 0.001 | 0.142 | 0.15 | 0 | 0.023 | 0.044 | 0.002 | MVAE |
| 0.075 | 0.259 | 0.006 | 0.003 | 0.013 | 417 | 1093 | 1.169 | 0.062 | 0.001 | 0.054 | 0.106 | 0.003 | 0.021 | 0.024 | 0.002 | NSGA-II |
| 0.085 | **0.239** | **0.01** | **0.003** | **0.01** | **254** | **1035** | **1.274** | **0.158** | **0.001** | **0.051** | **0.127** | **0.003** | **0.024** | **0.025** | **0.001** | **NSGA-II** |
| 0.087 | 0.314 | 0.008 | 0.002 | 0.013 | 319 | 1065 | 1.182 | 0.146 | 0.003 | 0.142 | 0.127 | 0 | 0.023 | 0.044 | 0.002 | MVAE |
| 0.062 | 0.249 | 0.006 | 0.003 | 0.014 | 376 | 1033 | 1.182 | 0.146 | 0.001 | 0.142 | 0.127 | 0 | 0.023 | 0.044 | 0.002 | NSGA-II |
| 0.091 | 0.279 | 0.012 | 0.003 | 0.014 | 362 | 1135 | 1.182 | 0.146 | 0.001 | 0.142 | 0.14 | 0.01 | 0.023 | 0.044 | 0.002 | MVAE |
| 0.076 | **0.241** | **0.007** | **0.002** | **0.013** | **414** | **971** | **1.47** | **0.149** | **0.002** | **0.004** | **0.086** | **0.002** | **0.026** | **0.032** | **0.001** | **NSGA-II** |
| 0.075 | 0.226 | 0.009 | 0.001 | 0.015 | 206 | 1147 | 1.46 | 0.146 | 0.002 | 0.189 | 0.15 | 0 | 0.023 | 0.044 | 0.002 | NSGA-II |
| 0.061 | 0.27 | 0.009 | 0.001 | 0.012 | 236 | 1100 | 1.541 | 0.088 | 0.002 | 0.004 | 0.124 | 0.001 | 0.018 | 0.021 | 0.002 | NSGA-II |
| 0.055 | 0.271 | 0.008 | 0.002 | 0.013 | 434 | 963 | 1.207 | 0.156 | 0.002 | 0.076 | 0.031 | 0.001 | 0.015 | 0.038 | 0.002 | NSGA-II |
| 0.082 | 0.301 | 0.006 | 0.004 | 0.011 | 448 | 1263 | 1.201 | 0.065 | 0.001 | 0.192 | 0.108 | 0.002 | 0.015 | 0.044 | 0 | NSGA-II |
| 0.08 | 0.225 | 0.006 | 0.002 | 0.013 | 328 | 1203 | 1.46 | 0.146 | 0.002 | 0.189 | 0.15 | 0 | 0.023 | 0.044 | 0.002 | NSGA-II |
| 0.082 | 0.244 | 0.007 | 0.002 | 0.014 | 445 | 1164 | 1.182 | 0.146 | 0.001 | 0.142 | 0.127 | 0 | 0.023 | 0.044 | 0.002 | NSGA-II |
| 0.053 | 0.251 | 0.012 | 0.002 | 0.015 | 290 | 1170 | 1.182 | 0.146 | 0.003 | 0.142 | 0.127 | 0 | 0.023 | 0.044 | 0.002 | MVAE |
| 0.092 | 0.302 | 0.01 | 0.003 | 0.014 | 345 | 1095 | 1.182 | 0.146 | 0.002 | 0.142 | 0.14 | 0 | 0.023 | 0.044 | 0.002 | MVAE |
| 0.075 | 0.216 | 0.008 | 0.001 | 0.015 | 228 | 1195 | 1.46 | 0.146 | 0.002 | 0.189 | 0.15 | 0 | 0.023 | 0.044 | 0.002 | NSGA-II |
| 0.055 | 0.295 | 0.01 | 0.002 | 0.011 | 427 | 1034 | 1.46 | 0.146 | 0.002 | 0.189 | 0.15 | 0 | 0.023 | 0.044 | 0.002 | NSGA-II |
| 0.079 | 0.238 | 0.007 | 0.003 | 0.01 | 196 | 1033 | 1.46 | 0.146 | 0.002 | 0.189 | 0.15 | 0 | 0.023 | 0.044 | 0.002 | NSGA-II |
| 0.09 | 0.305 | 0.009 | 0.002 | 0.014 | 342 | 1098 | 1.182 | 0.146 | 0.003 | 0.142 | 0.127 | 0 | 0.023 | 0.044 | 0.002 | MVAE |
| 0.091 | 0.297 | 0.011 | 0.003 | 0.014 | 350 | 1115 | 1.182 | 0.146 | 0.002 | 0.142 | 0.15 | 0 | 0.023 | 0.044 | 0.002 | MVAE |
| 0.084 | 0.321 | 0.007 | 0.002 | 0.013 | 311 | 1044 | 1.182 | 0.146 | 0.003 | 0.142 | 0.15 | 0 | 0.023 | 0.044 | 0.002 | MVAE |
| 0.079 | 0.273 | 0.009 | 0.002 | 0.01 | 312 | 1070 | 1.182 | 0.146 | 0.003 | 0.142 | 0.15 | 0 | 0.023 | 0.044 | 0.002 | MVAE |
| 0.07 | **0.272** | **0.007** | **0.003** | **0.011** | **449** | **1121** | **1.24** | **0.132** | **0.001** | **0.006** | **0.112** | **0.003** | **0.02** | **0.041** | **0.002** | **NSGA-II** |
| 0.09 | 0.273 | 0.013 | 0.004 | 0.014 | 382 | 1150 | 1.182 | 0.146 | 0.001 | 0.142 | 0.14 | 0 | 0.023 | 0.044 | 0.002 | MVAE |
| 0.088 | 0.315 | 0.008 | 0.002 | 0.014 | 323 | 1075 | 1.182 | 0.146 | 0.003 | 0.142 | 0.127 | 0 | 0.023 | 0.044 | 0.002 | MVAE |
| 0.086 | 0.289 | 0.015 | 0.001 | 0.016 | 332 | 1207 | 1.182 | 0.146 | 0.003 | 0.142 | 0.127 | 0 | 0.023 | 0.044 | 0.002 | MVAE |
| 0.08 | 0.323 | 0.006 | 0.002 | 0.013 | 299 | 1025 | 1.182 | 0.146 | 0.003 | 0.142 | 0.127 | 0 | 0.023 | 0.044 | 0.002 | MVAE |
| 0.089 | 0.255 | 0.01 | 0.002 | 0.011 | 297 | 1232 | 1.182 | 0.146 | 0.003 | 0.142 | 0.15 | 0 | 0.023 | 0.044 | 0.002 | MVAE |
| 0.088 | 0.249 | 0.01 | 0.002 | 0.011 | 296 | 1229 | 1.182 | 0.146 | 0.003 | 0.142 | 0.15 | 0 | 0.023 | 0.044 | 0.002 | MVAE |
| 0.067 | 0.231 | 0.015 | 0.003 | 0.011 | 367 | 976 | 1.182 | 0.146 | 0.001 | 0.142 | 0.127 | 0 | 0.023 | 0.044 | 0.002 | NSGA-II |
| 0.057 | 0.239 | 0.006 | 0.002 | 0.011 | 228 | 1123 | 1.46 | 0.146 | 0.002 | 0.189 | 0.15 | 0 | 0.023 | 0.044 | 0.002 | NSGA-II |
| 0.065 | 0.216 | 0.005 | 0.003 | 0.015 | 495 | 1268 | 1.182 | 0.146 | 0.001 | 0.142 | 0.127 | 0 | 0.023 | 0.044 | 0.002 | NSGA-II |
| 0.055 | **0.242** | **0.006** | **0.004** | **0.015** | **317** | **1102** | **1.377** | **0.142** | **0.001** | **0.014** | **0.066** | **0.002** | **0.018** | **0.036** | **0.001** | **NSGA-II** |
| 0.056 | 0.312 | 0.012 | 0.001 | 0.014 | 484 | 975 | 1.182 | 0.146 | 0.001 | 0.142 | 0.127 | 0 | 0.023 | 0.044 | 0.002 | NSGA-II |
| 0.074 | **0.261** | **0.006** | **0.002** | **0.014** | **332** | **1036** | **1.499** | **0.136** | **0.002** | **0.015** | **0.088** | **0.002** | **0.02** | **0.033** | **0.001** | **NSGA-II** |
| 0.086 | 0.315 | 0.008 | 0.002 | 0.013 | 316 | 1059 | 1.182 | 0.146 | 0.003 | 0.142 | 0.15 | 0 | 0.023 | 0.044 | 0.002 | MVAE |
| 0.054 | **0.24** | **0.006** | **0.003** | **0.015** | **319** | **1107** | **1.397** | **0.142** | **0.001** | **0.013** | **0.064** | **0.002** | **0.018** | **0.036** | **0.001** | **NSGA-II** |
| 0.083 | 0.217 | 0.009 | 0.001 | 0.012 | 225 | 1172 | 1.182 | 0.146 | 0.001 | 0.142 | 0.127 | 0 | 0.023 | 0.044 | 0.002 | NSGA-II |
| 0.09 | 0.295 | 0.01 | 0.003 | 0.014 | 348 | 1102 | 1.182 | 0.146 | 0.002 | 0.142 | 0.14 | 0 | 0.023 | 0.044 | 0.002 | MVAE |
| 0.056 | 0.214 | 0.011 | 0.002 | 0.015 | 280 | 1067 | 1.46 | 0.146 | 0.001 | 0.189 | 0.15 | 0 | 0.023 | 0.044 | 0.002 | MVAE |
| 0.085 | 0.301 | 0.014 | 0.001 | 0.015 | 318 | 1176 | 1.182 | 0.146 | 0.003 | 0.142 | 0.15 | 0 | 0.023 | 0.044 | 0.002 | MVAE |
| 0.08 | 0.322 | 0.006 | 0.002 | 0.013 | 294 | 1028 | 1.182 | 0.146 | 0.003 | 0.142 | 0.127 | 0.01 | 0.023 | 0.044 | 0.002 | MVAE |
| 0.088 | 0.309 | 0.009 | 0.002 | 0.013 | 329 | 1079 | 1.182 | 0.146 | 0.003 | 0.142 | 0.127 | 0 | 0.023 | 0.044 | 0.002 | MVAE |
| 0.082 | 0.314 | 0.006 | 0.003 | 0.014 | 436 | 1011 | 1.182 | 0.146 | 0.001 | 0.142 | 0.127 | 0 | 0.023 | 0.044 | 0.002 | NSGA-II |
| 0.054 | 0.277 | 0.008 | 0.002 | 0.013 | 434 | 963 | 1.207 | 0.156 | 0.002 | 0.076 | 0.031 | 0.001 | 0.015 | 0.037 | 0.002 | NSGA-II |
| 0.081 | 0.238 | 0.014 | 0.001 | 0.013 | 249 | 1171 | 1.182 | 0.146 | 0.001 | 0.142 | 0.127 | 0 | 0.023 | 0.044 | 0.002 | NSGA-II |
| 0.072 | 0.282 | 0.009 | 0.003 | 0.012 | 355 | 1130 | 1.182 | 0.146 | 0.001 | 0.142 | 0.127 | 0 | 0.023 | 0.044 | 0.002 | NSGA-II |
| 0.065 | 0.25 | 0.008 | 0.001 | 0.014 | 352 | 1167 | 1.456 | 0.063 | 0.002 | 0.087 | 0.098 | 0.003 | 0.026 | 0.043 | 0.003 | NSGA-II |

**Table S4**. The calculated Ae1 and Ae3 temperatures, and the evaluated precipitation reactions of (Ti,Nb)C and VC for five selected superior alloys.

|  | C | Si | P | S | Ti | Mn | Cu | Sn | Ni | Cr | Mo | V | Nb | Ca |
| --- | --- | --- | --- | --- | --- | --- | --- | --- | --- | --- | --- | --- | --- | --- |
| 1 | 0.093 | 0.301 | 0.010 | 0.002 | 0.014 | 1.182 | 0.146 | 0.003 | 0.142 | 0.127 | 0 | 0.023 | 0.044 | 0.002 |
| 2 | 0.084 | 0.226 | 0.006 | 0.029 | 0.013 | 1.182 | 0.146 | 0.003 | 0.142 | 0.127 | 0 | 0.023 | 0.044 | 0.002 |
| 3 | 0.073 | 0.248 | 0.008 | 0.004 | 0.014 | 1.460 | 0.146 | 0.001 | 0.189 | 0.150 | 0 | 0.023 | 0.044 | 0.002 |
| 4 | 0.085 | 0.240 | 0.010 | 0.003 | 0.010 | 1.182 | 0.146 | 0.003 | 0.051 | 0.127 | 0 | 0.024 | 0.025 | 0.001 |
| 5 | 0.072 | 0.282 | 0.009 | 0.003 | 0.012 | 1.182 | 0.146 | 0.001 | 0.142 | 0.127 | 0 | 0.023 | 0.044 | 0.002 |
|  | **Ae1**  **[℃]** | **Ae2**  **[℃]** | **Ti,Nb.C**  **(start)**  **[ ℃]** | **Ti,Nb.C**  **(end)**  **[℃]** | **VC**  **(start)**  **[℃]** | **VC**  **(end)**  **[℃]** | **Class** |  |  |  |  |  |  |  |
| 1 | 685 | 841 | 1214 | 341 | 741 | 358 | NSGA-II |  |  |  |  |  |  |  |
| 2 | 684 | 846 | 1203 | 360 | 739 | 370 | MVE |  |  |  |  |  |  |  |
| 3 | 669 | 833 | 1194 | 410 | 729 | 380 | MVE |  |  |  |  |  |  |  |
| 4 | 687 | 843 | 1163 | 354 | 745 | 402 | NSGA-II |  |  |  |  |  |  |  |
| 5 | 684 | 847 | 1188 | 370 | 738 | 390 | MVE |  |  |  |  |  |  |  |
